# Accuracy of Machine Learning Algorithms for the Diagnosis of Autism Spectrum Disorder: Systematic Review and Meta-Analysis of Brain Magnetic Resonance Imaging Studies

**DOI:** 10.2196/14108

**Published:** 2019-12-20

**Authors:** Sun Jae Moon, Jinseub Hwang, Rajesh Kana, John Torous, Jung Won Kim

**Affiliations:** 1 Ewha Womans University Mokdong Hospital Ewha Womans University Medical Center Seoul Republic of Korea; 2 Department of Computer Science and Statistics Daegu University Gyeongsangbuk-do Republic of Korea; 3 Department of Psychology University of Alabama at Tuscaloosa Tuscaloosa, AL United States; 4 Department of Psychiatry Beth Israel Deaconess Medical Center Harvard Medical School Boston, MA United States; 5 Department of Psychiatry and Behavioral Neurobiology University of Alabama at Birmingham Birmingham, AL United States

**Keywords:** autism spectrum disorder, machine learning, sensitivity and specificity, systematic review, meta-analysis

## Abstract

**Background:**

In the recent years, machine learning algorithms have been more widely and increasingly applied in biomedical fields. In particular, their application has been drawing more attention in the field of psychiatry, for instance, as diagnostic tests/tools for autism spectrum disorder (ASD). However, given their complexity and potential clinical implications, there is an ongoing need for further research on their accuracy.

**Objective:**

This study aimed to perform a systematic review and meta-analysis to summarize the available evidence for the accuracy of machine learning algorithms in diagnosing ASD.

**Methods:**

The following databases were searched on November 28, 2018: MEDLINE, EMBASE, CINAHL Complete (with Open Dissertations), PsycINFO, and Institute of Electrical and Electronics Engineers Xplore Digital Library. Studies that used a machine learning algorithm partially or fully for distinguishing individuals with ASD from control subjects and provided accuracy measures were included in our analysis. The bivariate random effects model was applied to the pooled data in a meta-analysis. A subgroup analysis was used to investigate and resolve the source of heterogeneity between studies. True-positive, false-positive, false-negative, and true-negative values from individual studies were used to calculate the pooled sensitivity and specificity values, draw Summary Receiver Operating Characteristics curves, and obtain the area under the curve (AUC) and partial AUC (pAUC).

**Results:**

A total of 43 studies were included for the final analysis, of which a meta-analysis was performed on 40 studies (53 samples with 12,128 participants). A structural magnetic resonance imaging (sMRI) subgroup meta-analysis (12 samples with 1776 participants) showed a sensitivity of 0.83 (95% CI 0.76-0.89), a specificity of 0.84 (95% CI 0.74-0.91), and AUC/pAUC of 0.90/0.83. A functional magnetic resonance imaging/deep neural network subgroup meta-analysis (5 samples with 1345 participants) showed a sensitivity of 0.69 (95% CI 0.62-0.75), specificity of 0.66 (95% CI 0.61-0.70), and AUC/pAUC of 0.71/0.67.

**Conclusions:**

The accuracy of machine learning algorithms for diagnosis of ASD was considered acceptable by few accuracy measures only in cases of sMRI use; however, given the many limitations indicated in our study, further well-designed studies are warranted to extend the potential use of machine learning algorithms to clinical settings.

**Trial Registration:**

PROSPERO CRD42018117779; https://www.crd.york.ac.uk/prospero/display_record.php?RecordID=117779

## Introduction

### Background

Autism spectrum disorder (ASD), behaviorally characterized by a deficit in social communication and rigidity in interest or behavior by both the Diagnostic and Statistical Manual of Mental Disorders-5 (DSM-5) and the International Statistical Classification of Diseases-11 (ICD-11), is believed to be a product of complex interactions between genetic and environmental factors [1-3]. The latest prevalence of ASD has been reported to be 1 in 59 children aged 8 years, based on the 2014 Center for Disease Control and Prevention (CDC) surveillance data [4], and 1 in 40 children aged 3-17 years, based on parental reports of the diagnosis in a national survey [5]. Despite the advancement of many biomarkers with potential in prediction or early detection of ASD (eg, structural magnetic resonance imaging [sMRI] or functional magnetic resonance imaging [fMRI]), a diagnosis is not made until the age of 4-5 years, on average [4,6].

Machine learning has been increasingly studied as a novel tool to enhance the accuracy of diagnosis and early detection of ASD [7]. Unlike traditional rule-based algorithms that allowed computers to generate answers with preprogramed rules, machine learning allows building of an algorithm that can learn, predict, and improve with experience, based on big data [3,8-10]. Psychiatric decision making is more sophisticated and difficult to characterize, compared with machine learning, although there are some common elements. Psychiatrists diagnose patients by observing their behaviors and registering all collected and collateral data into their (psychiatrists’) cognitive system as sensory input values (eg, voice and vision). Similarly, machine learning requires a series of steps, including preprocessing (eg, noise removal from data before input into an algorithm), segmentation, and feature extraction [7]. In particular, machine learning in the field of ASD diagnostics incorporates big data (eg, neuroimaging), making the input data immense and complex [11]. The application of machine learning algorithms in the field of neuroimaging often requires an extra process, such as *feature selection* that extracts key features from a complex dataset. In other words, key features are selected before the learning process, which is called *feature selection* [11].

### Objective

Currently, machine learning is widely applied to the field of bioinformatics, including genetics and imaging, and many applications require signal recognition and processing [12]. Machine learning algorithms are currently applied to the field of psychiatry in areas such as genomics, electroencephalogram (EEG), and neuroimaging. However, owing to the complex workflows implicated in machine learning itself, the accuracy of such algorithms is varied [8]. This study aimed to suggest an integrated estimate of the accuracy for use of machine learning algorithms in distinguishing individuals with ASD from control groups through systematic review and meta-analysis of the available studies.

## Methods

### Systematic Review

This systematic review and meta-analysis was conducted based on the Preferred Reporting Items for Systematic Reviews and Meta-Analyses for Diagnostic test accuracy [13]. The study protocol was written before initiation of the study and registered in the Prospective Register of Systematic Reviews database (trial registration: CRD42018117779).

### Data Sources and Search Strategy

MEDLINE, EMBASE, CINAHL Complete (with Open Dissertations), and PsycINFO were selected as core search databases, and the Institute of Electrical and Electronics Engineers (IEEE) Xplore Digital Library was added to maximize the sensitivity of the search. The IEEE Xplore Digital Library is a database created by the IEEE, the largest of its kind worldwide, and includes more than 1800 peer-reviewed conference proceedings. Default search filters provided by journals were not used. There was no restriction by publication type (eg, conference proceedings) or language. The initial search was conducted on November 28, 2018. The search strategy and query per search database are listed in Multimedia Appendix 1. The primary consideration for study inclusion was if machine learning was partially or fully applied in distinguishing individuals clinically diagnosed with ASD from controls and assess the accuracy of such applications. Multimedia Appendix 2 lists inclusion/exclusion criteria. An author (SM) retrieved the initial search results and removed duplicates by using the command *find duplicate* via a reference software (Endnote X9, Clarivate Analytics, Philadelphia, Pennsylvania. Subsequently, another author (JK) manually searched for and removed any residual duplicates. Finally, the studies were screened independently by two authors (SM and JK) by title, abstract, and keywords, after which the full texts of the selected studies were screened by two authors (SM and JK) by inclusion/exclusion criteria. If any discrepancy was found in the final selection, the two authors reached a consensus via discussion.

### Data Extraction

A data extraction form was created through discussion among the authors before the extraction process to suggest specific subgroups and coding processes (categorizing) for a meta-analysis (Multimedia Appendix 3). The process is provided in detail in Multimedia Appendix 4. General characteristics such as author, publication year, sample size, average age, gender ratio, and data characteristics were extracted from individual studies. Information regarding the reference standard used in individual studies and definitions of positive/negative disease (autism positive/control) and methodologies to distinguish individuals with autism from control group were collected. Specific methodologies used to process and classify data for use in machine learning algorithms were also recorded (Multimedia Appendices 3 and 4). All accuracy values were extracted, and true-positive / true-negative / false-positive / false-negative (TP/TN/FP/FN) values were calculated from individual studies for a meta-analysis. If the TP/TN/FP/FN values could not be calculated from the accuracy values provided in a study, an email was sent to the corresponding author to request raw data. If there was no response within 14 days, the study was not included in the meta-analysis. The extraction was performed independently by two authors (SM and JK). If there was any discrepancy in the extracted data, a consensus was reached by thorough discussion after repeating the same extraction process.

### Quality Assessment

Two authors (SM and JK) independently assessed the quality of individual studies based on the Quality Assessment of Diagnostic Accuracy Studies-2 (QUADAS-2). QUADAS-2 is a validated tool used to evaluate the quality of diagnostic accuracy studies by patient selection, index test, reference standard, and risk of bias (RoB) for internal validity and external validity for applicability concerns of individual studies [14]. There was no disagreement between authors in the assessment of patient selection and reference standard domain. The index test, also known as the target tool of our investigation in this study, is a machine learning algorithm. The target tool, the machine learning algorithm’s accuracy, is reported through a process called validation. However, when a study provided no information about the validation process, low RoB was assumed if independent datasets were used for training, building a model, and validation [15]. Otherwise, the level of RoB was determined by thoroughly reviewing the validation processes.

### Evidence Synthesis

In our meta-analysis, a bivariate random effects model was used to consider both within- and between-subject variability and threshold effect [16]. A Summary Receiver Operating Characteristics (SROC) curve was generated based on parameter estimates extracted from the bivariate random effects model [17]. The SROC curve was specified by pooled sensitivity, specificity point, 95% CIs, and prediction region. Area under the curve (AUC) and partial AUC (pAUC) were calculated based on the SROC curve [18]. Studies that were visually deviant from the 95% prediction region on the SROC curve were considered heterogeneous [19]. Attempts were made to resolve the heterogeneity by performing a subgroup analysis—generating individual SROC curves for subgroups (minimum 5 studies) [20]. If most studies were within the 95% prediction region on the SROC curves of the subgroups, the sample was determined to be homogeneous, and integrated sensitivity, specificity, and SROC curve results were provided. If any of the TP/FP/TN/FN value was 0, 0.5 was added to prevent zero cell count problem [21]. The TP/FP/TN/FN values were extracted or calculated from each independent sample in a study, and if multiple machine learning algorithms were applied to the same sample, an algorithm with the best accuracy (calculated as [TP+TN]/[TP+FP+TN+FN]) was selected for data extraction.

A meta-analysis was conducted via the mada package in R (version 3.4.3, R Core Team, Vienna, Austria), and statistical significance was expressed with 95% CIs. Publication bias was not assessed in our analysis, as there are currently no statistically adequate models in the field of meta-analysis of diagnostic test accuracy [22].

## Results

### Search, Selection, and General Characteristics

**Figure 1:**
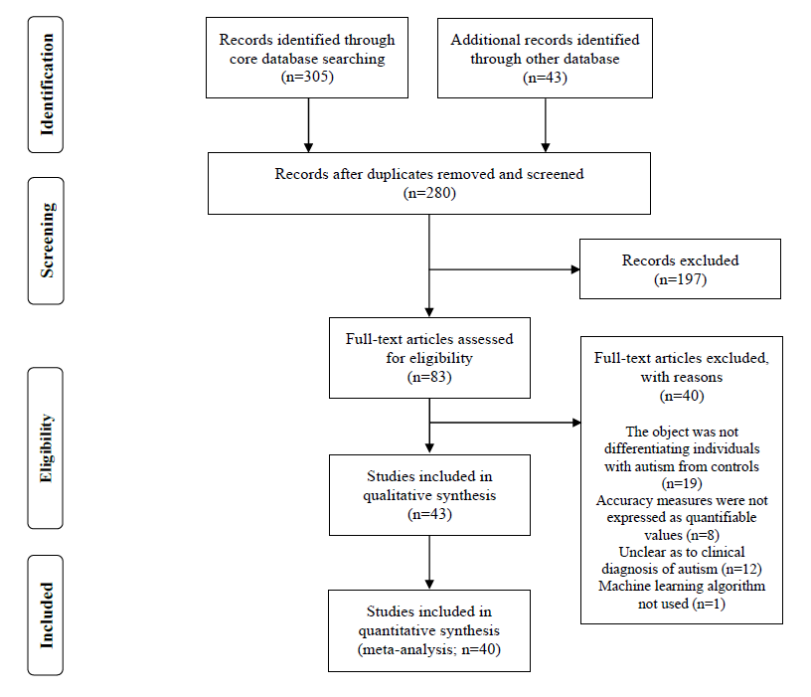
Flowchart for the literature screening and selection process.

After duplicate removal, of the 280 studies extracted from five databases and one additional database, 43 studies were selected, of which 40 studies were included in the meta-analysis. Figure 1 provides details according to the screening stage.

The publication years ranged from 2007 to 2018 for the final selection of 43 studies, of which 40 were journal articles and 3 were gray literature elements (eg, conference proceedings). A total of 10 studies used a public database that was available on the internet and open to anyone, 18 used a private sector database (eg, clinic and hospital), 3 used both public and private databases, and the remaining 12 used databases from others. Regarding the average age of the sample, 5 studies included adults, 22 studies included school-aged participants, 11 included preschool-aged participants, and the remaining 5 did not provide any information. For the machine learning algorithm, 20 studies used a support vector machine (SVM), 3 used a deep neural network (DNN), 13 used others, and the remaining 10 used and compared multiple algorithms. For prediction, 11 studies used sMRI features, 9 used fMRI features, 9 used behavior traits, 5 used biochemical features, 4 used EEG features, and the remaining 2 used text or voice features. For reference standards, 24 studies used DSM-IV, DSM-IV - Text Revision, or DSM-5; 10 used the Autism Diagnostic Observation Schedule (ADOS) or the Autism Diagnostic Interview (ADI); 2 used ICD; and the remaining 7 did not provide relevant information. For the validation methodology, 37 studies only used internal validation, 2 only used external validation, and 4 used both. The abovementioned information is summarized in Table 1, and the extracted raw data are presented in Multimedia Appendices 5 and 6.

**Table 1:** Characteristics of 43 studies for the systematic review and 53 samples for the meta-analysis.

Characteristics		Studies (n)^a^	Samples (n)^b^
**Publication type**			
	Journal article	40	50
	Letter, report, or conference proceeding	3	3
**Dataset type**			
	Private (hospital or clinic) dataset	18	21
	Public database	10	16
	Mixed (private and public) dataset	3	0
	Others or unknown	12	16
**Mean age of sample (years)**			
	Adults (≥18)	5	5
	School age (6-18)	22	27
	Preschool age (<6)	11	16
	Unknown	5	5
**Classification algorithm type**			
	Support vector machine	20	24
	Deep neural network	3	6
	Others^c^	13	23
	Mixed	10	0
**Predictor type**			
	Structural MRI^d^ features	11	14
	Functional MRI features^e^	9	13
	Behavior traits	9	14
	Biochemical features	5	7
	Electroencephalography features	4	3
	Text or voice	2	2
**Reference standard**			
	DSM^f^-IV (Text Revision) or DSM-5	24	28
	ADOS^g^ or ADI^h^	10	12
	ICD^i^	2	2
	Others or not otherwise specified	7	11
**Validation method**			
	Internal validation	36	46
	External validation	2	6
	Internal and external validation	4	0
	Others or not otherwise specified	1	1

^a^Number of studies for a given category (N=43 in total).

^b^Number of datasets used in studies (N=53 in total).

^c^Probabilistic neural network, decision tree, regression, ensemble, random forest, and fuzzy.

^d^MRI: magnetic resonance imaging.

^e^All studies used resting-state MRI images (one study used both resting state and task-related MRI images).

^f^DSM: Diagnostic and Statistical Manual of Mental Disorders.

^g^ADOS: Autism Diagnostic Observation Schedule.

^h^ADI: Autism Diagnostic Interview.

^i^ICD: International Statistical Classification of Diseases.

### Qualitative Assessment

**Figure 2:**
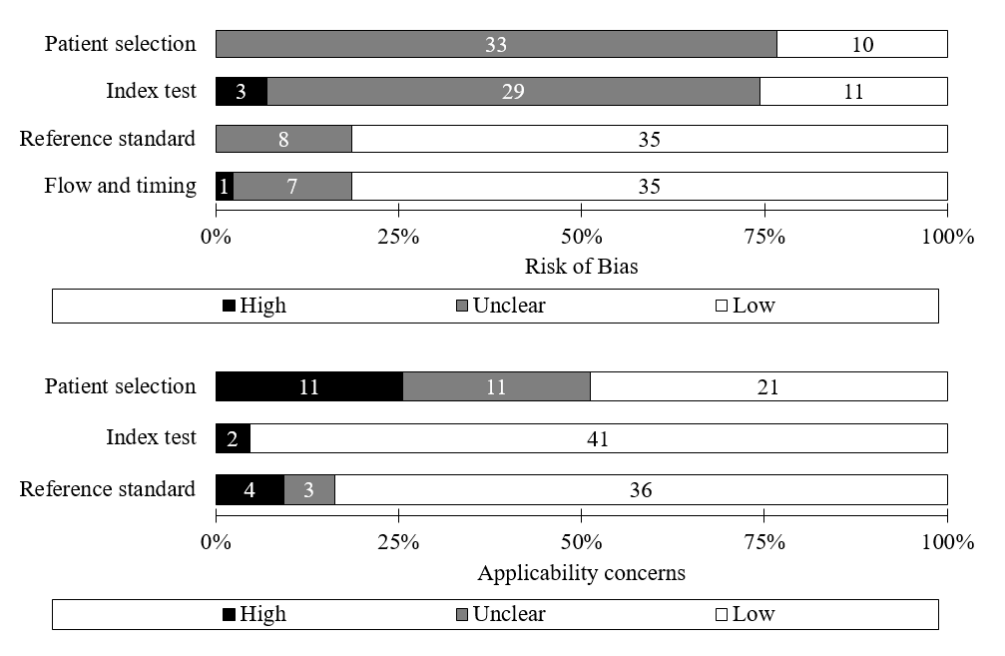
Risk of bias and applicability concern by domain in Quality Assessment of Diagnostic Accuracy Studies-2. Microsoft Excel was used.

Of the 43 studies in total, more than half were assessed to have an unclear RoB by patient selection domain (33 studies) and index test domain (29 studies). More than half were considered to have a low RoB by the total reference standard (35 studies) and flow and timing domains (35 studies). For applicability concern, about half (22 studies) were shown to have unclear or high-risk RoB by patient selection domain, whereas most were considered to have a low risk by index test (42 studies) and reference standard domain (36 studies). Qualitative assessment for all the individual studies is summarized in Multimedia Appendix 7, and the distribution is shown in Figure 2.

### Quantitative Analysis (Meta-Analysis)

**Figure 3:**
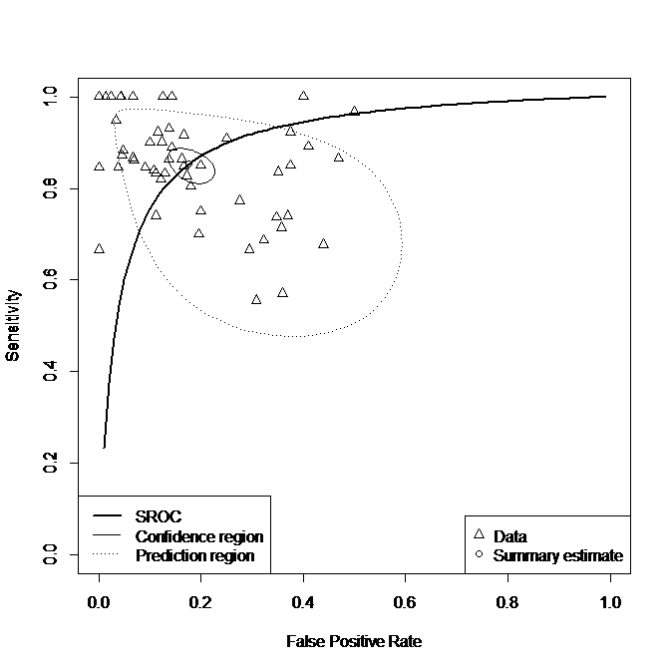
Summary Receiver Operating Characteristics curve for all 53 samples. Note that the confidence region is the 95% confidence region around the summary sensitivity and specificity points, and the prediction region is the 95% prediction of the true sensitivity and specificity interval for future observations. SROC: Summary Receiver Operating Characteristics.

Of the final selection of 43 studies, only 40, from which TP/FP/FN/TN values were extractable, were considered for the meta-analysis. A total of 53 independent samples were extracted from the 40 studies and included in the meta-analysis (Table 1). Of the 53 samples, 12,128 participants were inspected in the meta-analysis, with the total sensitivity and specificity ranging from 0.55 to 1.00 and 0.56 to 0.99, respectively. TP/FP/FN/TN, sensitivity, and specificity values for 53 individual samples are summarized in Multimedia Appendix 8, and visual distribution is provided as SROC in Figure 3. Of the 53 samples, 12 were found outside the 95% predictive region of the SROC curve, and therefore, there was heterogeneity between samples (Figure 3).

**Figure 4:**
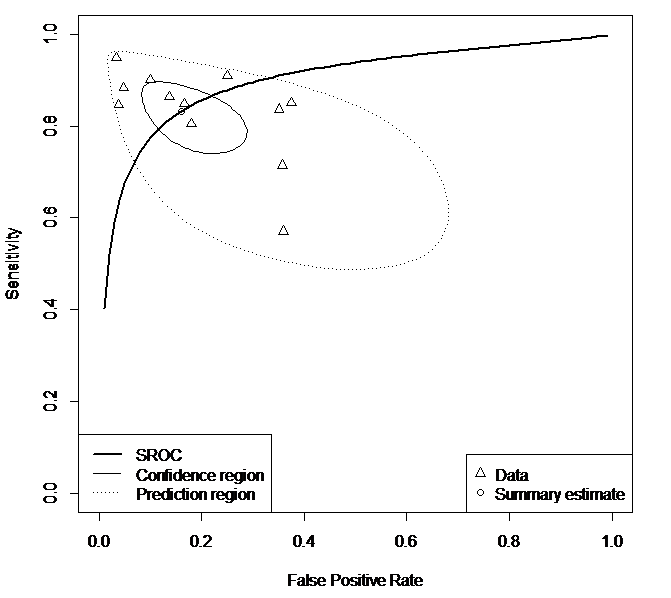
Summary Receiver Operating Characteristics curve for structural magnetic resonance imaging subgroup (12 samples). Note that the confidence region is the 95% confidence region around the summary sensitivity and specificity points, and the prediction region is the 95% prediction of the true sensitivity and specificity interval for future observations. SROC: Summary Receiver Operating Characteristics.

In an attempt to resolve this heterogeneity, a subgroup analysis was conducted with 19 variables that had been predefined and coded. For replicability, a raw data sheet listing the precodified variables is available in Multimedia Appendix 9. As a result, among 19 variables, *predictor* was the only one by which the heterogeneity could be partially resolved. Of the 53 samples, for the sMRI subgroup that used sMRI as predictors, all the 12 samples were found to be within the predictive region of the SROC curve, thus resolving the heterogeneity (Figure 4).

**Figure 5:**
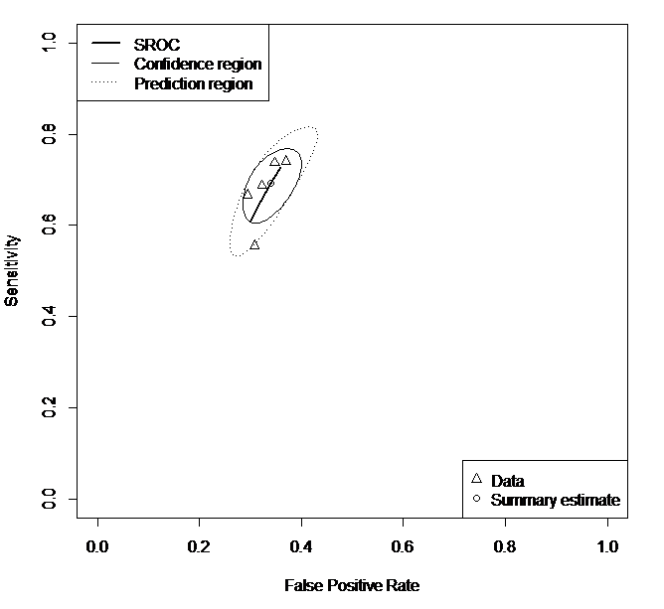


For the sMRI subgroup, the pooled sensitivity was 0.83 (95% CI 0.76-0.89), specificity was 0.84 (95% CI 0.74-0.91), and AUC/pAUC was 0.90/0.83. Meta-analysis was also attempted for the remaining subgroups, such as fMRI (15 samples), behavior traits (14 samples), and biochemical features (7 samples) subgroups, but the pooled sensitivity and specificity could not be provided owing to a significant degree of heterogeneity between samples: A few samples were shown to be far off the predictive region of the SROC curves (Multimedia Appendices 10-12). However, sub-subgroup meta-analysis using 5 samples that used fMRI as a predictor and DNN as a classifier allowed for the heterogeneity to be resolved and provided the pooled sensitivity of 0.69 (95% CI 0.62-0.75), specificity of 0.66 (95% CI 0.61-0.70), and AUC/pAUC of 0.71/0.67 (Figure 5).

Similarly, another sub-subgroup meta-analysis of six samples that used sMRI as a predictor and SVM as a classifier resolved the heterogeneity and resulted in a pooled sensitivity of 0.87 (95% CI 0.78-0.93), specificity of 0.87 (95% CI 0.71-0.95), and AUC/pAUC of 0.92/0.88 (Multimedia Appendix 12). Sensitivity and specificity values and types of classifiers used for samples of individual subgroups that used neuroimaging features (sMRI and fMRI subgroups) as predictors are provided in Table 2, and a forest plot is provided in Multimedia Appendix 13.

Summary Receiver Operating Characteristics curve for functional magnetic resonance imaging/deep neural network sub-subgroup (5 samples). Note that confidence region is the 95% confidence region around the summary sensitivity and specificity points, and the prediction region is the 95% prediction of the true sensitivity and specificity interval for future observations. SROC: Summary Receiver Operating Characteristics.

The sensitivity and specificity for the behavior traits (14 samples) subgroup ranged from 0.68 to 1.00 and 0.56 to 0.9, respectively. The sensitivity and specificity for the biochemical features (7 samples) subgroup ranged from 0.77 to 0.94 and 0.72 to 0.93, respectively. The sensitivity and specificity for the EEG subgroup (3 samples) ranged from 0.94 to 0.97 and 0.81 to 0.94, respectively. The results are summarized in Multimedia Appendix 8. Information for other measures not included in the meta-analysis is provided in Multimedia Appendix 14.

**Table 2:** Sensitivity and specificity of structural and functional magnetic resonance imaging subgroups.

MRI^a^ group		Sample size (n)	Sensitivity (95% CI)	Specificity (95% CI)
**Structural MRI subgroup**				
	Hazlett et al (2017) [23]	179	0.87 (0.72-0.95)	0.95 (0.90-0.97)
	Chaddad et al (2017)^b^ [24]	28	0.70 (0.45-0.87)	0.63 (0.39-0.83)
	Chaddad et al (2017)^c^ [24]	36	0.83 (0.63-0.94)	0.62 (0.39-0.81)
	Wee et al (2014) [25]	117	0.94 (0.85-0.98)	0.96 (0.88-0.99)
	Ecker et al (2010)^d^ [26]	44	0.85 (0.65-0.94)	0.85 (0.65-0.94)
	Ecker et al (2010)^d^ [27]	40	0.88 (0.68-0.96)	0.88 (0.68-0.96)
	Xiao et al (2017) [28]	85	0.80 (0.66-0.89)	0.81 (0.67-0.90)
	Katuwal et al (2015) [29]	734	0.57 (0.52-0.62)	0.64 (0.59-0.69)
	Jiao et al (2010) [30]	38	0.89 (0.71-0.97)	0.74 (0.50-0.89)
	Neeley et al (2007) [31]	57	0.84 (0.68-0.93)	0.82 (0.63-0.92)
	Kong et al (2019) [32]	182	0.84 (0.75-0.91)	0.96 (0.90-0.98)
	Shen et al (2018) [33]	236	0.83 (0.77-0.88)	0.65 (0.54-0.74)
	Subtotal by range and pooled estimate from meta-analysis	1776	0.57-0.94; 0.83 (0.76-0.89)	0.62-0.96; 0.84 (0.74-0.91)
**Functional MRI subgroup**				
	Li et al (2018)^b^ [34]	113	0.68 (0.54-0.80)	0.67 (0.55-0.78)
	Li et al (2018)^e^ [34]	75	0.55 (0.40-0.70)	0.69 (0.53-0.81)
	Li et al (2018)^f^ [34]	61	0.73 (0.58-0.84)	0.65 (0.45-0.80)
	Li et al (2018)^g^ [34]	61	0.66 (0.48-0.81)	0.70 (0.54-0.83)
	Heinsfeld et al (2018) [35]	1035	0.74 (0.70-0.78)	0.63 (0.59-0.67)
	Dekhil et al (2018) [36]	283	0.90 (0.83-0.94)	0.88 (0.82-0.92)
	Bernas et al (2018)^g^ [37]	30	0.89 (0.62-0.97)	0.81 (0.54-0.94)
	Mastrovito et al (2018) [38]	54	0.73 (0.55-0.86)	0.88 (0.71-0.95)
	Emerson et al (2017) [39]	59	0.82 (0.56-0.94)	0.99 (0.91-1.00)
	Price et al (2014) [40]	60	0.86 (0.69-0.94)	0.92 (0.77-0.98)
	Uddin et al (2013)^h^ [41]	40	0.74 (0.53-0.88)	0.79 (0.57-0.91)
	Uddin et al (2013)^i^ [41]	30	0.66 (0.42-0.84)	0.97 (0.76-1.00)
	Wang et al (2012) [42]	58	0.82 (0.65-0.92)	0.82 (0.65-0.92)
	Bernas et al (2018)^i^ [37]	24	0.81 (0.54-0.94)	0.87 (0.66-0.96)
	Lidaka (2015) [43]	640	0.92 (0.89-0.95)	0.88 (0.84-0.91)
	Subtotal	2623	0.55-0.92	0.63-0.99
Overall (sMRI^j^+fMRI^k^)		4399	0.55-0.94	0.62-0.99

^a^MRI: magnetic resonance imaging.

^b^Autism Brain Imaging Data Exchange-University of Michigan sample.

^c^Autism Brain Imaging Data Exchange-University of Pittsburgh sample.

^d^Same author years but different (independent) studies.

^e^Autism Brain Imaging Data Exchange-University of California Los Angeles sample.

^f^Autism Brain Imaging Data Exchange-University of Utah School of Medicine.

^g^Autism Brain Imaging Data Exchange-Katholieke Universiteit Leuven.

^h^National Database for Autism Research sample.

^i^Clinic sample.

^j^sMRI: structural magnetic resonance imaging.

^k^fMRI: functional magnetic resonance imaging.

## Discussion

### Principal Findings

On the basis of the meta-analysis in this study, the summary sensitivity and specificity of the accuracy for use of machine learning algorithms in ASD diagnosis are 0.83 (95% CI 0.76-0.89) and 0.84 (0.74-0.91), respectively, whereas the accuracy value based on AUC/pAUC is 0.90/0.83. On the basis of the opinion that the AUC/pAUC value is considered acceptable when above 0.7, both the AUC/pAUC values can be thought to be acceptable for the sMRI subgroup [44]. However, given the wide confidence interval for each summary sensitivity and specificity, the clinical usefulness of those values can be difficult to determine. In addition, precaution is warranted for interpreting the accuracy results, as the 95% predictive region is larger than the 95% CI region on the SROC curve, indicating a high degree of uncertainty for the pooled sensitivity and specificity calculated [19]. In addition, only one sample from the sMRI subgroup utilized an external validation method, where demographic characteristics of the training dataset were independent of those of the validation dataset. In other words, the rest of the samples in the sMRI subgroup built their validation datasets from participants who were similar to or the same as those recruited in the training datasets. Hence, those samples are believed to have high risks of overfitting, compromising the generalizability of machine learning models and overestimating the results of the meta-analysis of the sMRI subgroup [15].

Machine learning algorithms can be divided into supervised, unsupervised, or reinforcement learning by learning pattern [9]. SVM, for which subgroup analysis was performed for sMRI, is the oldest method of supervised learning, whereas DNN, for which subgroup analysis was conducted for fMRI, is the most advanced of the neural network methods (supervised learning), modeled after the mechanism of neurons [9]. On the contrary, the accuracy values for the fMRI subgroup using one of the latest machine learning algorithms, DNN, were found to be lower than those for the sMRI subgroup. This may, in part, be attributable to possible overestimation secondary to the overfitting in the sMRI subgroup. In addition, one of the studies in the fMRI/DNN sub-subgroup composed their dataset by recruiting over 1000 participants from various sites to minimize limitations such as overfitting in their analysis.

### Limitations

Our study has several limitations. Of the final selection of 43 studies, 33 did not provide clear information regarding the process of obtaining an original database or a recruiting training/validation dataset from the real clinical world, or raw data such as basic demographic characteristics of the participants before the input process, thus increasing the RoB in the patient selection processes. For example, more than half the finally selected studies did not match the samples for age or gender, and the number of images or signals per participant was not specified in most of the neuroimaging and EEG studies. Subgroups other than the sMRI subgroup included studies that used the same database, thus raising concerns for possible sample overlap, which was challenging to process statistically owing to the lack or absence of information on the patient selection process. If datasets overlapped and lowered the accuracy, the subgroup meta-analysis would have been underestimated and vice versa. In addition, behavior, EEG, and voice/text subgroups did not consist of enough studies to attempt to resolve the heterogeneity and provide pooled accuracy values. Furthermore, owing to the heterogeneity, summary accuracy values could not be obtained for adult (aged over 18 years), school-age (between 6 and 18 years), and preschool-age (less than 6 years) subgroups, thus limiting the ability to draw a conclusion on accuracy by age groups. Corresponding authors for individual studies with small and high T*P* values (ie, 100% accurate machine learning test) were reached out to, and one responded. Even if more had responded, to our knowledge, there would not have been any way to perform the aggregation.

### Comparison With Prior Work

To our knowledge, there is currently no study that has performed a systematic review and/or a meta-analysis on diagnostic test accuracy for the use of machine learning in diagnosing ASD and suggested its pooled estimate accuracies. In this analysis, many individual studies reported small TP and high TP (ie, 100% accurate machine learning test) and caused significant heterogeneity for a meta-analysis (see Figure 3). Authors resolved the heterogeneity by using subgroup analyses. As a result, individual studies with small and high T*P* values (ie, 100% accurate machine learning test) were barely included in fMRI and sMRI subgroup analyses, thereby resolving the heterogeneity and allowing conduct of the meta-analysis. Nevertheless, recommendations from our results may improve the quality of prospective studies using machine learning algorithms in ASD diagnosis. First, Standards for Reporting of Diagnostic Accuracy Studies (STARD) can guide machine learning diagnostic studies to enhance the reporting of patient selection processes. In addition, there is the comprehensive guideline for algorithm developers in terms of choosing an adequate predictive model for a target sample; setting the parameters, definition, or threshold; and minimizing errors such as overfitting and perfect separation [45]. Use of the STARD and other guidelines [45] would facilitate more transparent and comprehensive work in this space. Although not discussed in the studies included in our analysis, decision or running time for a machine learning algorithm in ASD diagnosis could become an important quality measure in the near future when these algorithms might be employed in a busy daily clinical practice.

### Conclusions

The accuracy of diagnosing ASD by machine learning algorithms was found to be acceptable by select accuracy measures only in studies that utilized sMRI. However, because of the high heterogeneity in the analyzed studies, it is impossible to draw a conclusion on any subgroups that used behavior traits or biochemical markers as predictors. There is a clear need for new studies with more comprehensive reporting of the selection process and dataset to draw a more accurate conclusion.

## Appendix

Multimedia Appendix 1 Search strategy and results.

Multimedia Appendix 2 Inclusion and exclusion criteria.

Multimedia Appendix 3 Data extraction form and detailed information for coding of subgroups.

Multimedia Appendix 4 Detailed process of data extraction.

Multimedia Appendix 5 General characteristics of studies (details).

Multimedia Appendix 6 Characteristics of the performance and validation condition (details).

Multimedia Appendix 7 Quality Assessment of Diagnostic Accuracy Studies ‐2 assessment of all studies.

Multimedia Appendix 8 True positive, false positive, false negative, true negative, sensitivity, and specificity of 53 study samples.

Multimedia Appendix 9 Precoded raw variable data sheet for subgroup analysis.

Multimedia Appendix 10 Summary Receiver Operating Characteristics curve for biochemical features subgroup (7 samples).

Multimedia Appendix 11 Summary Receiver Operating Characteristics curve for behavior trait subgroup (14 samples).

Multimedia Appendix 12 Summary Receiver Operating Characteristics curve for structural magnetic resonance imaging/support vector machine sub-subgroup (6 samples).

Multimedia Appendix 13 Forest plot of structural and functional magnetic resonance imaging subgroup.

Multimedia Appendix 14 Excluded accuracy indices for meta-analysis.

## Abbreviations

ASD: autism spectrum disorder
AUC: area under the curve
DNN: deep neural network
DSM: Diagnostic and Statistical Manual of Mental Disorders
EEG: electroencephalogram
fMRI: functional magnetic resonance imaging
FN: false negative
FP: false positive
ICD: International Statistical Classification of Diseases
IEEE: Institute of Electrical and Electronics Engineers
MRI: magnetic resonance imaging
pAUC: partial AUC
QUADAS-2: Quality Assessment of Diagnostic Accuracy Studies-2
RoB: risk of bias
sMRI: structural magnetic resonance imaging
SROC: Summary Receiver Operating Characteristics
STARD: Standards for Reporting of Diagnostic Accuracy Studies
SVM: support vector machine
TN: true negative
TP: true positive

## References

[1] Muhle RA, Reed HE, Stratigos KA, Veenstra-VanderWeele J (2018). The emerging clinical neuroscience of autism spectrum disorder: a review. JAMA Psychiatry.

[2] American Psychiatric Association (2013). Diagnostic and Statistical Manual of Mental Disorders, 5th Edition: DSM-5.

[3] (2018). ICD-11.

[4] Baio J, Wiggins L, Christensen DL, Maenner MJ, Daniels J, Warren Z, Kurzius-Spencer M, Zahorodny W, Rosenberg C, White T, Durkin MS, Imm P, Nikolaou L, Yeargin-Allsopp M, Lee L, Harrington R, Lopez M, Fitzgerald RT, Hewitt A, Pettygrove S, Constantino JN, Vehorn A, Shenouda J, Hall-Lande J, Braun K, Dowling NF (2018). Prevalence of autism spectrum disorder among children aged 8 years - autism and developmental disabilities monitoring network, 11 sites, United States, 2014. MMWR Surveill Summ.

[5] Kogan MD, Vladutiu CJ, Schieve LA, Ghandour RM, Blumberg SJ, Zablotsky B, Perrin JM, Shattuck P, Kuhlthau KA, Harwood RL, Lu MC (2018). The prevalence of parent-reported autism spectrum disorder among US children. Pediatrics.

[6] Zwaigenbaum L, Penner M (2018). Autism spectrum disorder: advances in diagnosis and evaluation. Br Med J.

[7] Thabtah F (2019). Machine learning in autistic spectrum disorder behavioral research: a review and ways forward. Inform Health Soc Care.

[8] Bzdok D, Meyer-Lindenberg A (2018). Machine learning for precision psychiatry: opportunities and challenges. Biol Psychiatry Cogn Neurosci Neuroimaging.

[9] Choy G, Khalilzadeh O, Michalski M, Do S, Samir AE, Pianykh OS, Geis JR, Pandharipande PV, Brink JA, Dreyer KJ (2018). Current applications and future impact of machine learning in radiology. Radiology.

[10] Jordan MI, Mitchell TM (2015). Machine learning: trends, perspectives, and prospects. Science.

[11] Kassraian-Fard P, Matthis C, Balsters JH, Maathuis MH, Wenderoth N (2016). Promises, pitfalls, and basic guidelines for applying machine learning classifiers to psychiatric imaging data, with autism as an example. Front Psychiatry.

[12] Obermeyer Z, Emanuel EJ (2016). Predicting the future - big data, machine learning, and clinical medicine. N Engl J Med.

[13] McInnes MD, Moher D, Thombs BD, McGrath TA, Bossuyt PM, Clifford T, Cohen JF, Deeks JJ, Gatsonis C, Hooft L, Hunt HA, Hyde CJ, Korevaar DA, Leeflang MM, Macaskill P, Reitsma JB, Rodin R, Rutjes AW, Salameh J, Stevens A, Takwoingi Y, Tonelli M, Weeks L, Whiting P, Willis BH, the PRISMA-DTA Group (2018). Preferred reporting items for a systematic review and meta-analysis of diagnostic test accuracy studies: the PRISMA-DTA statement. J Am Med Assoc.

[14] Whiting PF, Rutjes AW, Westwood ME, Mallett S, Deeks JJ, Reitsma JB, Leeflang MM, Sterne JA, Bossuyt PM, QUADAS-2 Group (2011). QUADAS-2: a revised tool for the quality assessment of diagnostic accuracy studies. Ann Intern Med.

[15] Park SH, Han K (2018). Methodologic guide for evaluating clinical performance and effect of artificial intelligence technology for medical diagnosis and prediction. Radiology.

[16] Reitsma JB, Glas AS, Rutjes AW, Scholten RJ, Bossuyt PM, Zwinderman AH (2005). Bivariate analysis of sensitivity and specificity produces informative summary measures in diagnostic reviews. J Clin Epidemiol.

[17] Arends LR, Hamza TH, van Houwelingen JC, Heijenbrok-Kal MH, Hunink MG, Stijnen T (2008). Bivariate random effects meta-analysis of ROC curves. Med Decis Making.

[18] Jones CM, Athanasiou T (2005). Summary receiver operating characteristic curve analysis techniques in the evaluation of diagnostic tests. Ann Thorac Surg.

[19] Lee J, Kim KW, Choi SH, Huh J, Park SH (2015). Systematic review and meta-analysis of studies evaluating diagnostic test accuracy: a practical review for clinical researchers-part ii. Statistical methods of meta-analysis. Korean J Radiol.

[20] Richardson M, Garner P, Donegan S (2019). Interpretation of subgroup analyses in systematic reviews: a tutorial. Clin Epidemiol Glob Health.

[21] Sweeting MJ, Sutton AJ, Lambert PC (2004). What to add to nothing? Use and avoidance of continuity corrections in meta-analysis of sparse data. Stat Med.

[22] Leeflang MM, Deeks JJ, Gatsonis C, Bossuyt PM, Cochrane Diagnostic Test Accuracy Working Group (2008). Systematic reviews of diagnostic test accuracy. Ann Intern Med.

[23] Hazlett HC, Gu H, Munsell BC, Kim SH, Styner M, Wolff JJ, Elison JT, Swanson MR, Zhu H, Botteron KN, Collins DL, Constantino JN, Dager SR, Estes AM, Evans AC, Fonov VS, Gerig G, Kostopoulos P, McKinstry RC, Pandey J, Paterson S, Pruett JR, Schultz RT, Shaw DW, Zwaigenbaum L, Piven J, IBIS Network, Clinical Sites, Data Coordinating Center, Image Processing Core, Statistical Analysis (2017). Early brain development in infants at high risk for autism spectrum disorder. Nature.

[24] Chaddad A, Desrosiers C, Hassan L, Tanougast C (2017). Hippocampus and amygdala radiomic biomarkers for the study of autism spectrum disorder. BMC Neurosci.

[25] Wee C, Wang L, Shi F, Yap P, Shen D (2014). Diagnosis of autism spectrum disorders using regional and interregional morphological features. Hum Brain Mapp.

[26] Ecker C, Rocha-Rego V, Johnston P, Mourao-Miranda J, Marquand A, Daly EM, Brammer MJ, Murphy C, Murphy DG, MRC AIMS Consortium (2010). Investigating the predictive value of whole-brain structural MR scans in autism: a pattern classification approach. Neuroimage.

[27] Ecker C, Marquand A, Mourão-Miranda J, Johnston P, Daly EM, Brammer MJ, Maltezos S, Murphy CM, Robertson D, Williams SC, Murphy DG (2010). Describing the brain in autism in five dimensions--magnetic resonance imaging-assisted diagnosis of autism spectrum disorder using a multiparameter classification approach. J Neurosci.

[28] Xiao X, Fang H, Wu J, Xiao C, Xiao T, Qian L, Liang F, Xiao Z, Chu KK, Ke X (2017). Diagnostic model generated by MRI-derived brain features in toddlers with autism spectrum disorder. Autism Res.

[29] Katuwal GJ, Cahill ND, Baum SA, Michael AM (2015). The predictive power of structural MRI in autism diagnosis. Conf Proc IEEE Eng Med Biol Soc.

[30] Jiao Y, Chen R, Ke X, Chu K, Lu Z, Herskovits EH (2010). Predictive models of autism spectrum disorder based on brain regional cortical thickness. Neuroimage.

[31] Neeley ES, Bigler ED, Krasny L, Ozonoff S, McMahon W, Lainhart JE (2007). Quantitative temporal lobe differences: autism distinguished from controls using classification and regression tree analysis. Brain Dev.

[32] Kong Y, Gao J, Xu Y, Pan Y, Wang J, Liu J (2019). Classification of autism spectrum disorder by combining brain connectivity and deep neural network classifier. Neurocomputing.

[33] Shen MD, Nordahl CW, Li DD, Lee A, Angkustsiri K, Emerson RW, Rogers SJ, Ozonoff S, Amaral DD (2018). Extra-axial cerebrospinal fluid in high-risk and normal-risk children with autism aged 2-4 years: a case-control study. Lancet Psychiatry.

[34] Li H, Parikh NA, He L (2018). A novel transfer learning approach to enhance deep neural network classification of brain functional connectomes. Front Neurosci.

[35] Heinsfeld AS, Franco AR, Craddock RC, Buchweitz A, Meneguzzi F (2018). Identification of autism spectrum disorder using deep learning and the ABIDE dataset. Neuroimage Clin.

[36] Dekhil O, Hajjdiab H, Shalaby A, Ali MT, Ayinde B, Switala A, Elshamekh A, Ghazal M, Keynton R, Barnes G, El-Baz A (2018). Using resting state functional MRI to build a personalized autism diagnosis system. PLoS One.

[37] Bernas A, Aldenkamp AP, Zinger S (2018). Wavelet coherence-based classifier: a resting-state functional MRI study on neurodynamics in adolescents with high-functioning autism. Comput Methods Programs Biomed.

[38] Mastrovito D, Hanson C, Hanson SJ (2018). Differences in atypical resting-state effective connectivity distinguish autism from schizophrenia. Neuroimage Clin.

[39] Emerson RW, Adams C, Nishino T, Hazlett HC, Wolff JJ, Zwaigenbaum L, Constantino JN, Shen MD, Swanson MR, Elison JT, Kandala S, Estes AM, Botteron KN, Collins L, Dager SR, Evans AC, Gerig G, Gu H, McKinstry RC, Paterson S, Schultz RT, Styner M, Schlaggar BL, Pruett JR, Piven J, IBIS Network (2017). Functional neuroimaging of high-risk 6-month-old infants predicts a diagnosis of autism at 24 months of age. Sci Transl Med.

[40] Price T, Wee CY, Gao W, Shen D (2014). Multiple-network classification of childhood autism using functional connectivity dynamics. Med Image Comput Comput Assist Interv.

[41] Uddin LQ, Supekar K, Lynch CJ, Khouzam A, Phillips J, Feinstein C, Ryali S, Menon V (2013). Salience network-based classification and prediction of symptom severity in children with autism. JAMA Psychiatry.

[42] Wang H, Chen C, Fushing H (2012). Extracting multiscale pattern information of fMRI based functional brain connectivity with application on classification of autism spectrum disorders. PLoS One.

[43] Iidaka T (2015). Resting state functional magnetic resonance imaging and neural network classified autism and control. Cortex.

[44] Mandrekar JN (2010). Receiver operating characteristic curve in diagnostic test assessment. J Thorac Oncol.

[45] Luo W, Phung D, Tran T, Gupta S, Rana S, Karmakar C, Shilton A, Yearwood J, Dimitrova N, Ho TB, Venkatesh S, Berk M (2016). Guidelines for developing and reporting machine learning predictive models in biomedical research: a multidisciplinary view. J Med Internet Res.

