# Supplementary material for: Accuracy of Machine Learning Algorithms for the Diagnosis of Autism Spectrum Disorder: Systematic Review and Meta-Analysis of Brain Magnetic Resonance Imaging Studies
Source: JMIR Ment Health. 2019 Dec 20;6(12):e14108. doi: 10.2196/14108 (PMC6942187; doi:10.2196/14108)
Supplement: Multimedia Appendix 1 [file mental_v6i12e14108_app1.pdf]

Multimedia Appendix 1. Search strategy and results.

**Database: MEDLINE & Embase[a]**		
#1	'autism'/exp OR ‘autism-spectrum-disorder*’ OR ‘autism*’ OR ‘autistic*’ OR ‘autism-infantil*’ OR ‘infantile-autis*’ OR ‘child-development-disorder*’ OR ‘pervasive-childhood-autis*’ OR ‘classical-autis*’ OR ‘Kanner-syndrom*’ OR ‘pervasive-developmental-disorder*’ OR ‘typical-autis*’ OR ‘primary-autis*’ OR ‘secondary-autis*’ OR ‘Asperger*’ OR ‘Rett*’	84,863
#2	'machine learning'/exp OR ‘machine-learning*’ OR ‘deep-learning*’ OR ‘regression-algorithm*’ OR ‘ordinary-least-squares-regression*’ OR ‘stepwise-regression*’ OR ‘multivariate-adaptive-regression-splines*’ OR ‘locally-estimated-scatterplot-smoothing*’ OR ‘instance-based*’ OR ‘nearest-neighbor*’ OR ‘learning-vector-quantization*’ OR ‘self-organizing-map*’ OR ‘locally-weighted-learning*’ OR ‘regularization-algorithm*’ OR ‘ridge-regression*’ OR ‘least-absolute-shrinkage-and-selection-operator*’ OR ‘least-absolute-shrinkage-selection-operator*’ OR ‘elastic-net*’ OR ‘least-angle-regression*’ OR ‘decision-tree*’ OR ‘classification-and-regression-tree*’ OR ‘classification-regression-tree*’ OR ‘iterative-dichotomiser*’ OR ‘chi-squared-automatic-interaction-detection*’ OR ‘decision-stump*’ OR ‘Bayesian*’ OR ‘Bayes*’ OR ‘clustering-algorithm*’ OR ‘k-means*’ OR ‘k-medians*’ OR ‘expectation-maximization*’ OR ‘hierarchical-clustering*’ OR ‘association-rule-learning-algorithm*’ OR ‘Apriori*’ OR ‘Eclat*’ OR ‘neural-network*’ OR ‘perceptron*’ OR ‘back-propagation*’ OR ‘Hopfield-network*’ OR ‘radial-basis-function-network*’ OR ‘deep-Boltzman-machine*’ OR ‘belief-network*’ OR ‘auto-encoder*’ OR ‘dimensionality-reduction-algorithm*’ OR ‘ensemble-algorithm*’ OR ’bootstrapped-aggregation*’ OR ‘Adaboost*’ OR ‘Stacked-generalization*’ OR ‘gradient-boosting-machine*’ OR ‘gradient-boosted-regression-tree*’ OR ‘random-forest*’ OR ‘support-vector*’ OR ‘Fuzzy*’ OR ‘Markov*’ OR ‘case-based-reasoning*’ OR ‘simulated-annealing*’ OR ‘inductive-logical-program*’ OR ‘genetic-algorithm*’ OR automatic-speech-recognition* OR part-of-speech-tagging*	319,952
#3	'area under the curve'/mj OR ‘sensitivity and specificity’/mj OR ‘Youden-index*’ OR ‘diagnostic-odds-ratio*’ OR ‘area-under-the-curve*’ OR ‘area-under-curve*’ OR ‘sensitivity*’ OR ‘specificity*’ OR ‘positive-predictive-value*’ OR ‘negative-predictive-value*’ OR ‘true-positive*’ OR ‘false-positive*’ OR ‘true-negative*’ OR ‘false-negative*’	1,857,013
#4	#1 AND #2 AND #3	186
**Database: CINAHL Complete & OpenDissertations**		
#1	(MH "Autistic Disorder") OR (MH "Asperger Syndrome") OR autism-spectrum-disorder* OR autism* OR autistic* OR autism-infantil* OR infantile-autis* OR child-development-disorder* OR pervasive-childhood-autis* OR classical-autis* OR Kanner-syndrom* OR pervasive-developmental-disorder* OR typical-autis* OR primary-autis* OR secondary-autis* OR Asperger* OR Rett*	30,712
#2	(MM "Machine Learning") OR machine-learning* OR deep-learning* OR regression-algorithm* OR ordinary-least-squares-regression* OR stepwise-regression* OR multivariate-adaptive-regression-splines* OR locally-estimated-scatterplot-smoothing* OR instance-based* OR nearest-neighbor* OR learning-vector-quantization* OR self-organizing-map* OR locally-weighted-learning* OR regularization-algorithm* OR ridge-regression* OR least-absolute-shrinkage-and-selection-operator* OR least-absolute-shrinkage-selection-operator* OR elastic-net* OR least-angle-regression* OR decision-tree* OR classification-and-regression-tree* OR classification-regression-tree* OR iterative-dichotomiser* OR chi-squared-automatic-interaction-detection* OR decision-stump* OR Bayesian* OR Bayes* OR clustering-algorithm* OR k-means* OR k-medians* OR expectation-maximization* OR hierarchical-clustering* OR association-rule-learning-algorithm* OR Apriori* OR Eclat* OR neural-network* OR perceptron* OR back-propagation* OR Hopfield-network* OR radial-basis-function-network* OR deep-Boltzman-machine* OR belief-network* OR auto-encoder* OR dimensionality-	44,992

	reduction-algorithm* OR ensemble-algorithm* OR bootstrapped-aggregation* OR Adaboost* OR Stacked-generalization* OR gradient-boosting-machine* OR gradient-boosted-regression-tree* OR random-forest* OR support-vector* OR Fuzzy* OR Markov* OR case-based-reasoning* OR simulated-annealing* OR inductive-logical-program* OR genetic-algorithm* OR automatic-speech-recognition* OR part-of-speech-tagging*	
#3	(MM "Sensitivity and Specificity") OR Youden-index* OR diagnostic-odds-ratio* OR area-under-the-curve* OR area-under-curve* OR sensitivity* OR specificity* OR positive-predictive-value* OR negative-predictive -value* OR true-positive* OR false-positive* OR true-negative* OR false-negative*	209,082
#4	#1 AND #2 AND #3	22
**Database: PsycINFO**		
#1	MAINSUBJECT.EXACT.EXPLODE("Autism Spectrum Disorders") OR 'autism-spectrum-disorder*' OR 'autism*' OR 'autistic*' OR 'autism-infantil*' OR 'infantile-autis*' OR 'child-development-disorder*' OR 'pervasive-childhood-autis*' OR 'classical-autis*' OR 'Kanner-syndrom*' OR 'pervasive-developmental-disorder*' OR 'typical-autis*' OR 'primary-autis*' OR 'secondary-autis*' OR 'Asperger*' OR 'Rett*'	74,701
#2	MAINSUBJECT.EXACT.EXPLODE("Machine Learning") OR 'machine-learning*' OR 'deep-learning*' OR 'regression-algorithm*' OR 'ordinary-least-squares-regression*' OR 'stepwise-regression*' OR 'multivariate-adaptive-regression-splines*' OR 'locally-estimated-scatterplot-smoothing*' OR 'instance-based*' OR 'nearest-neighbor*' OR 'learning-vector-quantization*' OR 'self-organizing-map*' OR 'locally-weighted-learning*' OR 'regularization-algorithm*' OR 'ridge-regression*' OR 'least-absolute-shrinkage-and-selection-operator*' OR 'least-absolute-shrinkage-selection-operator*' OR 'elastic-net*' OR 'least-angle-regression*' OR 'decision-tree*' OR 'classification-and-regression-tree*' OR 'classification-regression-tree*' OR 'iterative-dichotomiser*' OR 'chi-squared-automatic-interaction-detection*' OR 'decision-stump*' OR 'Bayesian*' OR 'Bayes*' OR 'clustering-algorithm*' OR 'k-means*' OR 'k-medians*' OR 'expectation-maximization*' OR 'hierarchical-clustering*' OR 'association-rule-learning-algorithm*' OR 'Apriori*' OR 'Eclat*' OR 'neural-network*' OR 'perceptron*' OR 'back-propagation*' OR 'Hopfield-network*' OR 'radial-basis-function-network*' OR 'deep-Boltzman-machine*' OR 'belief-network*' OR 'auto-encoder*' OR 'dimensionality-reduction-algorithm*' OR 'ensemble-algorithm*' OR 'bootstrapped-aggregation*' OR 'Adaboost*' OR 'Stacked-generalization*' OR 'gradient-boosting-machine*' OR 'gradient-boosted-regression-tree*' OR 'random-forest*' OR 'support-vector*' OR 'Fuzzy*' OR 'Markov*' OR 'case-based-reasoning*' OR 'simulated-annealing*' OR 'inductive-logical-program*' OR 'genetic-algorithm*' OR 'automatic-speech-recognition*' OR 'part-of-speech-tagging*'	67,945
#3	MAINSUBJECT.EXACT.EXPLODE("Test Sensitivity") OR MAINSUBJECT.EXACT.EXPLODE("Test Specificity") OR 'Youden-index*' OR 'diagnostic-odds-ratio*' OR 'area-under-the-curve*' OR 'area-under-curve*' OR 'sensitivity*' OR 'specificity*' OR 'positive-predictive-value*' OR 'negative-predictive-value*' OR 'true-positive*' OR 'false-positive*' OR 'true-negative*' OR 'false-negative*'	142,358
#4	#1 AND #2 AND #3	97
**Database: IEEE (Institute of Electrical and Electronics Engineers) Xplore digital library**		
	(autism* OR autistic* OR infantile autism OR child development disorder OR pervasive childhood autism OR Kanner syndrome OR pervasive developmental disorder OR primary autism OR secondary autism OR Asperger OR Rett) AND (learn* OR train* OR validat* OR learning OR training OR validating OR machine OR deep OR support vector OR neural network OR auto encoder OR belief network OR perceptron OR clustering algorithm OR random forest OR decision tree OR Fuzzy OR Bayesian OR Bayes OR automatic speech recognition OR Markov OR nearest neighbor) AND (Youden index OR diagnostic odds ratio OR area under the curve OR area under curve OR sensitivity OR specificity OR positive predictive	43

	value OR negative predictive value OR true positive OR false positive OR true negative OR false negative)

[a] Embase®, an integrated database of Embase and MEDLINE, by Elsevier® was used.
