# Supplementary material for: Accuracy of Machine Learning Algorithms for the Diagnosis of Autism Spectrum Disorder: Systematic Review and Meta-Analysis of Brain Magnetic Resonance Imaging Studies
Source: JMIR Ment Health. 2019 Dec 20;6(12):e14108. doi: 10.2196/14108 (PMC6942187; doi:10.2196/14108)
Supplement: Multimedia Appendix 2 [file mental_v6i12e14108_app2.pdf]

Multimedia Appendix 2. Inclusion and exclusion criteria.

**Inclusion criteria**
(a) the object of the study was to differentiate individuals clinically diagnosed with ASD from controls (e.g., neurotypical individuals or individuals with another neurodevelopmental disorder)<br>(b) algorithms, suggested as machine learning in the study, included appropriate apparatus, such as learning or training, aimed at seeking optimal answers<br>(c) such algorithms also included process of validation, in addition to training or learning<br>(d) MRI (e.g., functional, structural, etc.) was used in diagnostic imaging studies<br>(e) validation and/or accuracy measure were expressed as quantifiable values
**Exclusion criteria**
(a) the study included individuals diagnosed with ASD non-clinically or individuals at high risk for or suspected of ASD (without diagnostic confirmation)<br>(b) algorithms following a determined flow (e.g., rule-base algorithm), instead of searching for optimal answers<br>(c) training, learning, and/or validation process were not explained clearly or distinguished from each other<br>(d) non-human subjects (e.g., animals) were studied
