# Supplementary material for: Accuracy of Machine Learning Algorithms for the Diagnosis of Autism Spectrum Disorder: Systematic Review and Meta-Analysis of Brain Magnetic Resonance Imaging Studies
Source: JMIR Ment Health. 2019 Dec 20;6(12):e14108. doi: 10.2196/14108 (PMC6942187; doi:10.2196/14108)
Supplement: Multimedia Appendix 3 [file mental_v6i12e14108_app3.pdf]

**Multimedia Appendix 3. Data extraction form and detailed information for coding of subgroups.**

| Domain (number, name) | Extracted items | Initial coding (categorizing) | Subgroup (number, name) | Subgroup coding for meta-analysis or quality assessment |
|---|---|---|---|---|
| D1, primary information domain | First author | N/A | N/A | N/A |
| | Publication year | N/A | N/A | N/A |
| | Publication type | Research article, Letter, Brief communication (report), Conference proceeding, Dissertation, Others | D1-S1, Publication type | Research article (Journal)/others subgroup |
| D2, participations or dataset domain | Data subset | Training dataset | N/A | N/A |
| | | Internal validation (cross or split validation), external validation dataset | D2-S1, Validation dataset-1 | Internal/external/unknown subgroup |
| | | | D2-S2, Validation dataset-2 | Internal-cross/internal-split/external/unknown subgroup |
| | Dataset type (for validation) | Public, Private, Others | D2-S3, Dataset type | Public/Private/Others or unknown subgroup |
| | Number of sample | Per patients | N/A | N/A |
| | | Per cases (images, genes etc.) | N/A | N/A |
| | Mean age | < 6 years, 6 to 18 years, > 18 years | D2-S4, Age (6/18) | < 6 /6 to 18/> 18 years/ unknown subgroup |
| | | | D2-S5, Age (6) | < 6/> 6 years/ unknown subgroup |
| | | | D2-S6, Age (18) | < 18/> 18 years/ unknown subgroup |
| | Infant study | Infant study or not | D2-S7, Infant study | Infant study/not infant study/unknown subgroup |
| | Gender distribution | Male or female only, mixed or unknown | D2-S8, Gender distribution | Male only/female only/mixed/unknown subgroup |
| | Age or gender matched | Matched or not | D2-S9, Age or gender matched | Matched/not-Matched/unknown subgroup |
| | ASD subtype | Autistic disorder, Asperger's syndrome, Rett Syndrome, pervasive developmental disorder | N/A | N/A |

| | | | | |
|---|---|---|---|---|
| | | not otherwise specified (PDD-NOS) | | |
| | Psychiatric function | Intelligence, verbal ability etc. | - | - |
| | Quality parameters | Slice thickness, calibration data etc. | - | - |
| D3 (performance and validation condition domain) | Acquisition or pre-processing | Yes or no | D3-S1, Acquisition | Acquisition (Y)/ Acquisition (N) subgroup |
| | Segmentation | Manually, not machine learning algorithm based, machine learning algorithm based segmented | D3-S2, Segmentation | Manually/not machine learning algorithm based/machine learning algorithm based/Not applicable/unknown subgroup |
| | Extraction | Filter, wrapper, embedded, manually or others | D3-S3, Extraction | Filter/wrapper/embedded/manually/others subgroup |
| | Predictor (for classification) | Structural MRI, functional MRI, biochemical markers, behavior assessment, voice, electroencephalography (EEG), others | D3-S4, Predictor | Structural MRI/functional MRI/biochemical markers/behavior assessment/voice/EEG/text subgroup |
| | Classification type | Support vector machine (SVM), neural network, decision tree, regression, ensemble, random forest, fuzzy etc. | D3-S5, Classifier tech-tree-1 | SVM/deep-neural network(DNN)/ Probabilistic neural network/decision tree/regression/ensemble/random forest/fuzzy subgroup |
| | | Supervised, unsupervised, reinforce, mixed or others | D3-S6, Classifier tech-tree-2 | Supervised/unsupervised/others subgroup |
| | Reference standard | DSM or others | D3-S7, Reference standard | DSM/others subgroup |
| | Disease positive | Autistic disorder, Asperger’s syndrome, Rett Syndrome, pervasive developmental disorder not otherwise specified (PDD-NOS) | D3-S8, Autism characters | ASD/others subgroup |
| | Disease negative | Control (not-otherwise specified), typically developing, developing delayed, other psychiatric disorders etc. | D3-S9, Control characters | Typically developing/Others subgroup |
| D4 (accuracy indices domain) | Accuracy indices | True-positive/true-negative/false-positive/false-negative | D4S1 to D4S4 | TP/FP/FN/TN |
| | | Area Under the Curve, diagnostic odds ratio, | N/A | N/A |

| | |
|---|---|
| | Youden's J index etc. |

Note: N/A, Not Applicable.
