# Supplementary material for: Accuracy of Machine Learning Algorithms for the Diagnosis of Autism Spectrum Disorder: Systematic Review and Meta-Analysis of Brain Magnetic Resonance Imaging Studies
Source: JMIR Ment Health. 2019 Dec 20;6(12):e14108. doi: 10.2196/14108 (PMC6942187; doi:10.2196/14108)
Supplement: Multimedia Appendix 4 [file mental_v6i12e14108_app4.docx]

**Multimedia Appendix 4. Detailed process of data extraction.**

A list of data extraction items was developed based on the previously submitted protocol (see Prospective Register of Systematic Reviews database, CRD42018117779). The data extraction items could be broadly grouped into four domains (D1 to D4): D1) basic information of individual studies, D2) participations (or dataset), D3) performance and validation condition, and D4) accuracy indices.

For D1 (basic information of individual studies domain), the name of the first author, publication year and type were extracted, of which publication type (D1-S1) was selected as the only variable for subgroup analysis.

D2 (participations or dataset domain) was divided into training dataset and validation dataset. Training dataset is not known to have a sub-category, whereas validation dataset can be further divided by types into internal validation, whose sample originates from the same sample as the training dataset, and external validation, whose sample is composed of interpedently sampled data (D2-S1), and internal-split validation, which uses a sample that has been separated from the original dataset for the purpose of validation, and internal-cross validation, which repeats validation process over a sample that is left out of the training dataset (D2-S2) [1]. For machine learning algorithms, there are two types of dataset: 1) public – open and available to anyone (e.g., via internet) and has encrypted sensitive information, 2) private – requires permission from an organization, institution, or a hospital, and has sometimes encrypted sensitive information. Other types of dataset (e.g., trials) are coded as miscellaneous (D2-S3) [2]. For sample size, the number of target condition (autism positive and negative) and the number of extracted data per participant (e.g., the number of brain MRI images or genes) were extracted separately, in order to see if input data had been extracted repeatedly from a single participant. Participants’ average age and gender were also extracted. Participants’ average age was divided and coded into pre-school age (below six years old), school age (six to 18 years old), adults (at or above 18 years old) (D2-S4). To rule out cases where studies may not be evenly distributed over different subgroups, participants’ average age was also tried to be divided and coded into ‘below six years old, at or above six years old (D2-S5)’ and ‘below 18 years old, at or above 18 years old (D2-S6)’. In particular, a group of studies targeting infants was coded separately (D2-S7). Participants’ gender was categorized by ASD versus control, which information was coded depending on whether a group had male- or female-only or mixed (D2-S8). Also, whether or not the age matches with the gender (yes or no) was extracted and coded (D2-S9). Whether individuals with autism spectrum disorder (ASD) or certain subtype(s) of ASD were included was assessed, which information was used in quality assessment of applicability concern for patient selection. Also, measurable cognitive functions (e.g., intelligence, verbal ability) were extracted; however, could not be coded due to lack or absence of consensus between studies on definition of high versus low functioning. Last, parameters indicative of dataset quality were extracted. Machine learning algorithms (in particular, imaging studies) are known to be influenced by the quality of input data [3]. Quality parameters, such as resolution - e.g., slice thickness in imaging studies, presence or absence of calibration in biochemical marker studies – were extracted but could not be coded due to various ways of reporting; hence, were not included in the meta-analysis.

For D3 (performance and validation condition domain), subgroups were determined by diagnostic workflow [4], by which machine learning algorithms processed input data. First, for acquisition or pre-processing, during which input data are simply processed without modification, only the information whether the process was performed or not was coded, due to various ways of reported methodologies by studies (D3-S1). Second, for segmentation, by which input data are broken into smaller units or regions, whether segmentation was performed or not, as well as, whether machine learning algorithms were used or not were considered in coding (D3-S2). Third, detailed methods of extracting input data for machine learning algorithms were categorized. Studies that used feature selection are classified into filter, wrapper, and embedded, depending on the degree to which they match with the structure of feature selection search and classification model [5]. Cases other than described above were coded as ‘others’, and those extracted by humans were coded as ‘manually’ (D3-S3). Fourth, the last step of machine learning, classification, was extracted and coded by the type of predictors (D3-S4) and algorithms (D3-S5). In particular, machine learning algorithms were divided by the type of its task into supervised, unsupervised, and mixed or others (D3-S6) [6]. Fifth, depending on reference standard to confirm the diagnosis of autism, whether Diagnostic and Statistical Manual of Mental Disorders (DSM) was used or not was coded (D3-S7). Based on the reference standard, definitions for disease (autism) positive versus negative were considered in coding. For disease positive (autism), whether the definition of ASD was based on DSM or not (D3-S8), and for disease negative, whether control was defined as typically developing or other than ASD were coded (D3-S9).

For D4 (accuracy indices domain), all accuracy values were extracted, in particular true-positive/true-negative/false-positive/false-negative (TP/TN/FP/FN) values for meta-analysis (D4-S1 to D4-S4), while miscellaneous values, such as Area Under the Curve (AUC) or diagnostic odds ratio, Youden’s J index, were extracted separately. The following is the data extraction form and detailed information for coding of subgroups (see Multimedia Appendix 3).

**References**

1. Park SH, Han K. Methodologic Guide for Evaluating Clinical Performance and Effect of Artificial Intelligence Technology for Medical Diagnosis and Prediction. Radiology. 2018 Mar;286(3):800-9. PMID: 29309734. doi: 10.1148/radiol.2017171920.
2. Gillies RJ, Kinahan PE, Hricak H. Radiomics: Images Are More than Pictures, They Are Data. Radiology. 2016 Feb;278(2):563-77. PMID: 26579733. doi: 10.1148/radiol.2015151169.
3. Lee JG, Jun S, Cho YW, Lee H, Kim GB, Seo JB, et al. Deep Learning in Medical Imaging: General Overview. Korean journal of radiology. 2017 Jul-Aug;18(4):570-84. PMID: 28670152. doi: 10.3348/kjr.2017.18.4.570.
4. Thabtah F. Machine learning in autistic spectrum disorder behavioral research: A review and ways forward. Informatics for health & social care. 2018 Feb 13:1-20. PMID: 29436887. doi: 10.1080/17538157.2017.1399132.
5. Saeys Y, Inza I, Larranaga P. A review of feature selection techniques in bioinformatics. Bioinformatics. 2007 Oct 1;23(19):2507-17. PMID: 17720704. doi: 10.1093/bioinformatics/btm344.
6. Choy G, Khalilzadeh O, Michalski M, Do S, Samir AE, Pianykh OS, et al. Current Applications and Future Impact of Machine Learning in Radiology. Radiology. 2018 Aug;288(2):318-28. PMID: 29944078. doi: 10.1148/radiol.2018171820.
