# Supplementary material for: Accuracy of Machine Learning Algorithms for the Diagnosis of Autism Spectrum Disorder: Systematic Review and Meta-Analysis of Brain Magnetic Resonance Imaging Studies
Source: JMIR Ment Health. 2019 Dec 20;6(12):e14108. doi: 10.2196/14108 (PMC6942187; doi:10.2196/14108)
Supplement: Multimedia Appendix 5 [file mental_v6i12e14108_app5.pdf]

Multimedia Appendix 5. General characteristics of studies (details).

	Publication type	Dataset characters		Number of sample, n (= autism positive + negative)		Sociodemographic characters of sample (Autism +, -)			Disease characters (autism)		Data quality parameters	Accuracy indices
		Subset	Type (name)	Per patients	Per cases (images, genes etc.)	Age, years (SD)	Gender (F/M)	Others	Subtype	Other psychiatric function		
Kong 2018	Research article	Training, internal validation (cross)	Public (ABIDE I-NYU)	182 (= 78 + 104)	-	14.5 (5.3), 15.9 (5.0)	10/68, 26/78	-	ASD		sMRI, 3 Tesla, eye tracker applied	Sens, Sepc, Acc and AUC
		External validation	Public (ABIDE I-KKI)	-	-	-	-	-	ASD	-		
Wan 2018	Research article	Training, internal validation (cross)	Private (hospital)	74 (= 37 + 37)	74 (= 37 +37), per eye tracker records	4.6 (0.7), 4.8 (0.4)	4/33, 10/27	-	ASD	-	Eye tracker, 250 Hz (sampling frequency), 0.03° (spatial resolution)	Sens, Sepc and Acc
Shen 2018	Research article	Training	Private (hospital)	-	-	-	-	-	-	-	sMRI, 3 Tesla, calibration phantom applied	Sens, Sepc, PPV and Acc
		External validation	Private (hospital)	236 (= 159 + 77)	-	3.1 (0.5), 3.0 (0.4)	27/132, 28/49	-	ASD	Verbal ability* [55.7 (27.0), 107.8 (12.3)]; Non-verbal ability [70.7 (19.0) 105.1 (14.7)]	sMRI, 3 Tesla, calibration phantom applied	
Sharma 2018	Research article	Training, internal validation	-	80 (= 40 + 40)	-	3.6, 3.7	11/29, 12/28	-	ASD	-	N/A	Sens, Sepc, and Acc
Mastrovito 2018	Research article	Training	Mixed (public and private)	64 (= 37 + 27)	-	-	-	-	ASD	-	Rs-fMRI	Sens, Sepc, and Acc
		Internal validation	Mixed (public and private)	-	-	-	-	-	ASD	-	Rs-fMRI	
		Training	Public (ABIDE I-USM)	64 (= 37 + 27)	-	22.7 (7.7), 21.4 (7.6)	0/37, 0/27	-	ASD	-	Rs-fMRI, verbal instructions prior to and during the scan session	Sens, Sepc, and Acc
		External validation	Private (hospital)	54 (= 27 + 27)	-	39.6 (15.1), 37.6 (16.1)	0/27, 0/27	-	ASD	-	Rs-fMRI, 3 Tesla, goggles, headphones and a response device applied	
Li 2018	Research article	Training	Public (ABIDE I, UM, UCLA, USM, LEUVEN)	411 (for test group of UM), 437 (UCLA), 453 (USM), 442 (LEUVEN)	-	-	-	-	-	-	fMRI	
		Internal validation (cross)	Public (ABIDE I-UM)	113 (= 48 + 65)	-	13.8 (2.0), 15.0 (3.7)	9/39, 16/49	-	ASD	IQ 107.6 (17.3), 109.0 (9.5)	fMRI	Sens, Sepc, Acc and AUC
			Public (ABIDE I-UCLA)	75 (= 36 + 39)	-	13.3 (3.0), 13.2 (1.8)	2/34, 6/33	-	ASD	IQ 102.4 (12.8), 106.4 (10.4)	fMRI	Sens, Sepc, Acc and AUC
			Public (ABIDE I-USM)	61 (= 38 + 23)	-	24.6 (9.0), 22.3 (7.9)	0/38, 0/23	-	ASD	99.7 (17.3), 115.5 (15.6)	fMRI	Sens, Sepc, Acc and AUC
			Public (ABIDE I-LEUVEN)	61 (= 27 + 34)	-	18.0 (5.0), 18.2 (5.1)	2/25, 5/34	-	ASD	109.4 (13.1), 114.8 (12.9)	fMRI	Sens, Sepc, Acc and AUC

Heunis 2018	Research article	Training and internal validation (cross)	Private (hospital)	62 (= 16 + 46) and 14 (= 7 + 7) for age matched subsample	4,802 segments	4.0, 7.9 and 4.0, 3.9 for age matched subsample	1:3, 1:1.4 and 1:2.5, 1:1.3 for age matched subsample	-	ASD	-	EEG, Biologic recording system (256–512 Hz sampling rate, 0.1–100 Hz band pass range) or a Natus Neuroworks system (200 Hz sampling rate, 0.1–100 Hz band pass range)	Sens, Sepc and Acc
Heinsfeld 2018	Research article	Training and internal validation (cross)	Public (ABIDE I from 17 sites)	1,035 (= 505 + 530)	-	10.0 to 27.4, 10.0 to 28.0 from 17 sites	62/443, 95/435	-	ASD	-	Rs-fMRI, Mean Framewise Displacement rated for quality measure	Sens, Sepc and Acc
Dekhil 2018	Research article	Training and internal validation (cross)	Public (NDAR)	283 (= 112 + 171)s	-	13.1 (2.5), 12.9 (3.1) for female, 12.9 (3.0), 13.0 (2.8) for male	56/67, 85/75	-	ASD	100 to 103 for ASD, 107 to 111 for TD (DAS-II subscale mean score)	T1 weighted sMRI and a rs-fMRI, 3 Tesla	Sens, Sepc, Acc and AUC
Castelhano 2018	Research article	Training and internal validation (cross)	Private (hospital)	20 (= 10 + 10)	-	23.5 (7.0), 23.1 (5.6)	0/10, 0/10	-	ASD	IQ 95.2 (14.3), 98.7 (14.0)	EEG Presentation 14.0., NuAmps 40 Channel Quick-Cap EEG system	Sens, Sepc, Acc and AUC
Bernas 2018	Research article	Training or internal validation (cross)	Private (hospital)	24 (= 12 + 12)	-	15.5 (1.0), 14.4 (1.3)	-	-	ASD	IQ 116.7 (5.0), 113.2 (7.8)	Rs-fMRI, 3 Tesla	
		Training and internal validation (cross)	Public (ABIDE I-Leuven)	30 (= 12 + 18)	-	13.7 (1.2), 14.4 (1.6)	-	-	ASD	IQ 101.9 (14.7), 106.1 (8.6)	Rs-fMRI, 3 Tesla, instructed to lie with their eyes closed, to think of nothing but not to fall asleep	Sens, Sepc, PPV, NPV and Acc
Askari 2018	Research article	Training, internal validation (cross)	Private (hospital)	183 (= 89 + 94)	-	9.7 (2.3), 9.3 (1.9)	36/53, 33/61	-	ASD (Asperger's/PDD-NOS/AD 46/20/23)	IQ 110.4 (9.7), 112.3 (7.9)	EEG, wireless Emotiv Epoch headset	Sens, Sepc and Acc
Anwar 2018	Research article	Training, internal validation (cross)	Private (hospital)	69 (= 38 + 31)	69 (= 37 + 32) for urine sample, 48 (= 27 + 21) for plasma sample	7.6 (2.0), 8.6 (2.0)	9/29, 8/23	-	ASD (early/ regressive / mixed 22/6/10)	IQ by PEP-3 normal or borderline/ mild/moderate/severe 11/3/12/12	LC-MS/MS, with correction for autohydrolysis of hydrolytic enzymes	Sens, Sepc, PPV, NPV, +/- LR, Acc and AUC
Abbas 2018	Research article	Training	Others (multiple repositories of survey)	2,686 (= 2,299 + 387)	-	621 (= 414 + 207) for < 4 years, 2,065 (= 1,885 + 180) for ≥ 4 years	-	-	Autism condition	-	N/A	
		External validation (cross)	Others (multiple	162 (= 121 + 41)	162 (= 121 + 41)	105 (= 84 + 21) for < 4 years, 57	-	-	Autism condition	-	N/A	Sens, Sepc, and AUC

			repositories of survey)			(= 37 + 20) for ≥ 4 years						
Xiao 2017	Research article	Training, internal validation (cross)	Private (hospital)	85 (= 46 + 39)	-	2.3 (0.3), 2.3 (0.3)	5/41, 5/34	-	PDD	DQ 73.0 (30.0), 69.0 (23.0)	sMRI, 3 Tesla, slice thickness 1.33 mm	Sens, Sepc, Acc, AUC and Mcc
Nakai 2017	Research article	Training, internal validation (cross)	Private (hospital)	81 (= 30 + 51)	-	7.3 (1.3), 7.4 (1.7)	8/22, 21/30	-	ASD	IQ 68.2 (16.9) for ASD group	N/A	Sens, Sepc, FPR, FNR and Acc
Oh 2017	Research article	Training and internal validation (cross)	Public (Gene Expression Omnibus)	42 (= 21 + 21) for unsupervised learning	-	26.7 (5.5), 27.0 (5.5)	4/17, 4/17	-	ASD	IQ (WAIS) 91.9 (21.6) for ASD group	Whole Human Genome Microarray GPL6480	Sens, Sepc, PPV, NPV and Acc
		Training or internal validation (randomly split)	Public (Gene Expression Omnibus)	26 (= 13 + 13) for training and 16 (= 8 + 8) for validation for supervised learning	-	-	-	-	ASD	-	Whole Human Genome Microarray GPL6480	Sens, Sepc, and Acc
Hazlett 2017	Letter	Training and internal validation (cross)	Private (hospital)	179 (= 34 + 145)	-	2.0	-	-	ASD	-	sMRI, 3 Tesla, slice thickness = 2 mm with quality controlled with phantom	Sens, Sepc, PPV, NPV and Acc
Emerson 2017	Report	Training	Private (hospital)	58	Exactly 150 noncensored frames were used	N/A	N/A	-	ASD	MSEL-fine motor 24.1 (1.0), 23.8 (0.3), receptive language 24.4 (0.8), 26.5 (0.5)	fMRI, 3 Tesla, by IBIS imaging protocol	Sens, Sepc, and Acc
		Internal validation (cross)	Private (hospital)	59 (= 11 + 48)	-	2.0	0/11, 18/30		ASD	-	fMRI, 3 Tesla, by IBIS imaging protocol	Sens, Sepc, and Acc
Chaddad 2017	Research article	Training, internal validation (cross)	Public (ABIDE I-UM)	28 (= 14 + 14)	-	12.9, 14.0	8/6, 8/6		ASD	-	sMRI, 3 Tesla	Sens, Sepc, and Acc
			Public (ABIDE I-Pitt)	36 (= 20 + 16)	-	17.0, 16.5	3/17, 2/14		ASD	-		Sens, Sepc, and Acc
Bosl 2017	Research article	Training, internal validation (cross)	Private (hospital)	41 (= 18 + 23)	6,000 points	8.8 (1.9), 8.6 (1.4)	2/16, 2/21		ASD	-	EEG, high density 128-channel Geodesic Sensor Net	Sens, Sepc, and Acc
Maenner 2016	Research article	Training	Others (ADDM 2008, Georgia)	1,162 (= 601 + 561)	5,396 evaluations	-	-	-	PDDNOS + AD	-	N/A	-
		Validation	Others (ADDM 2010, Georgia)	1,450 (= 754 + 696)	9,811 evaluations	3.3 to 4.4 for total sample	71.8 to 83.7% male for total sample	-	PDDNOS + AD	14.9 to 35.4 for IQ ≤ 70 (%)	N/A	Sens, Spec, PPV, NPV, Cohen's Kappa and AUC
Liu 2016	Research article	Training, Internal validation (cross)	Private (hospital)	87 (= 29 + 58)	-	7.9 (1.5) for ASD group, 7.9 (1.4) / 5.8 (1.0) for age/ability matched TD group	4/25, 8/50	-	ASD	NVIQ 22.3 (10.8) for ASD group, 29.9 (10.0) / 22.3 (7.9) for age/ability matched TD group	Eye tracker (sample rate: 60 Hz)	Sens, Spec, Acc, AUC

Li 2016	Research article	Total sample (randomly split to group 1 to 2)	Private (hospital)	47 (= 25 + 22)		9.3 (1.4), 9.5 (1.6)	7/18, 4/18	-	ASD	RSPM-IQ 91.0 (15.0), 106.0 (12.0)	fNIRS, 780 nm, 805 nm and 830 nm laser	-
		Group-1, Training	Private (hospital)	23 (= 12 + 11)	-	-	-	-	ASD	-		-
		Group-2, internal validation (split)	Private (hospital)	24 (= 13 + 11)	-	-	-	-	ASD	-		Sens, Spec and PPV
Duda 2016	Research article	Training, internal validation (cross)	Others (SSC, AGRE and AC dataset)	2,925 (= 2,775 + 150)	-	-	15.6%/83.9 % for ASD group, 37.3%/62.0 % for ADHD group	-	ASD	-	N/A	AUC
Cohen 2016	Research article	Total sample (randomly split into group 1 to 3)	Others (IBR BAR, INFANT, Q- GLO and ICD)	660 (= 535 + 125)	-	5.7 (2.9), 4.6	16.0% female for ASD group 25 to 43.0% female for control group	-	ASD	-	N/A	LR
		Group-1, training and internal		338 (= 272 + 66)	-	-	-	-	ASD	-	N/A	Sens, Spec, PPV and NPV
		Group-2, internal (split)		163 (= 132 + 31)	-	-	-	-	ASD	-	N/A	Sens, Spec, PPV and NPV
		Group-3, internal (split)		159 (= 130 + 29)	-	-	-	-	ASD	-	N/A	Sens, Spec, PPV and NPV
Bone 2016	Research article	Training, internal (cross)	Unknown (Balanced Independent Dataset)	567 (= 389 +178) for age above 10 group, 319 (= 238 + 81) for age below 10 group	-	7.1 (1.7), 7.1 (1.7) for age above 10 group, 14.7 (5.2), 16.1 (8.8) for age below 10 group	19.0% female for ASD group 33.1% female for control group	-	ASD	IQ 92.6 (22.3), 92.7 (20.5) for age above 10 group 95.5 (23.8), 92.1 (23.7) for age below 10 group	N/A	Sens, Spec and UAR
Prampar o 2015	Research article	Training and internal (cross)	Private (clinics and community)	142 (= 87 + 55)	-	2.3 (0.7), 1.5 to 2.0	0/87, 0/55	-	ASD	-	Microarray, quality criteria were used to exclude low-quality arrays	Sens, Spec, Acc and AUC
		Internal (split)	Private (clinics and community)	73 (= 44 + 29)	-	2.3 (0.8), 1.2 to 1.6	0/44, 0/29	-	ASD	-		Sens, Spec, Acc and AUC
Katuwal 2015	Conference proceeding	Training, internal (cross)	Public (ABIDE, 15 sites)	734 (= 361 + 373)	-	-	-	-	ASD	-	sMRI	Sens, Spec, Acc, AUC
Lidaka 2015	Research article	Training, internal (cross)	Public (ABIDE, 12 sites)	640 (= 312 + 328)	82 to 250 (mean = 179) images for each subject	13.2 (3.1), 12.9 (3.0)	39/273, 61/267	-	ASD	-	Rs-fMRI, 3T, general experimental procedure used	Sens, Spec, PPV, NPV and Acc,
Crippa 2015	Research article	Training, internal (cross)	Private (clinics and community)	30 (= 15 + 15)	-	3.5 (7.7), 2.6 (5.2)	3/12, 2/13	-	ASD	-	Optoelectronic system (infrared-motion analysis cameras at 60 Hz)	Sens, Spec and Acc
West 2014	Research article	Total sample (randomly split)	Private (clinics and community)	82 (= 52 + 30)	-	5.4 (0.8), 5.6 (1.0)	13.3% female for ASD group	-	AD	IQ 67.5 (17.7), 114.3 (10.8)	LC-HRMS and further confirmed by MS	-

							21.2% female for control group					
		Training, internal (cross)		61 (= 39 +22)	-	-	-	-	AD	-		Acc and AUC
		Internal (split)		21 (= 13 +8)	-	-	-	-	AD	-		Sens, Spec, Acc and AUC
Wee 2014	Research article	Training, internal (cross)	Public (NDAR)	117 (= 58 +59)	-	10.7 to 11.5, 11.0 (4.0)	13/45, 22.0% female/67.8 % male for control group (10.2% missing)	-	ASD	-	sMRI, slice thickness = 0.82 to 1.33 mm	Sens, Spec and Acc, AUC
Price 2014	Research article	Training, internal (cross)	Public (ABIDE-NYU)	60 (= 30 + 30)	-	9.8 (1.4), 9.7 (1.6)	-	-	ASD	-	Rs-fMRI, 3 Tesla	Sens, Spec, PPV, NPV, Acc
Uddin 2013	Research article	Training, internal (cross)	Private (clinics and community)	40 (= 20 + 20)	-	9.96(1.59), 9.95(1.60)	4/16, 4/16	-	ASD	112.6 (17.8), 112.1 (15.4)	Rs-fMRI, 3 Tesla, slice 4.0mm, head cushion applied, EEand T1-weighted sMRI	Sens, Spec, PPV, NPV, and Acc
		External	Public (NDAR)	30 (= 15 + 15)	-	10.2 (1.65) for TD group	4/11 for TD group	-	ASD	-	-	
Wang 2012	Research article	Training, internal (cross)	Others (institute and community)	58 (= 29 + 29)	-	-	5/24, 5/24	-	ASD	-	Resting state and task-related fMRI	Sens and Spec
Wall 2012 (1)	Research article	Training, internal (cross)	Others (AGRE, AC)	627 (= 612 + 15)	-	-	-	-	Autism	-	N/A	Sens, Spec and Acc
Wall (2) 2012	Research article	Training, internal (cross)	Others (AGRE)	966 (= 891 + 75)	-	8.1, 9.2	-	-	Autism	-	N/A	Sens, Spec and Acc
		External	Others (AC)	1,659 (= 1,654 + 5)	-	8.8, 9.8	-	-	Autism	-	N/A	Sens, Spec and Acc
		External	Others (SSC)	334 (= 322 + 12)	-	8.5, 9.5	-	-	Autism	-	N/A	Sens, Spec and Acc
Calderoni 2012	Research article	Training, internal (cross)	Private (clinical institute)	76 (= 38 + 38)	-	4.4 (1.5), 4.4 (1.6)	38/0, 38/0	-	ASD	-	3D-sMRI, 1.5T, slice thickness = 1.1mm	AUC
Jiao 2010	Research article	Training, internal (cross)	Private (hospital)	38 (= 22 + 16)	-	9.2 (2.1), 10.0 (1.9)	3/19, 3/13	-	ASD	WISC IQ 102.0 (22.4), 107.5 (20.7)	sMRI, 1.5T, slice thickness = 2.0mm	Sens, Spec, Acc and AUC
Ecker 2010 (1)	Research article	Training, internal (cross)	Others (institute and community)	44 (= 22 + 22)	-	27.0 (7.0), 28.0 (7.0)	0/22, 0/22	-	ASD	IQ 104.0 (15.0) 111.0 (10.0)	sMRI, 3T, slice thickness = 1.1mm with semi-automated quality control procedure	Sens, Spec and Acc
Ecker 2010 (2)	Research article	Training, internal (cross)	Others (institute and community)	40 (= 20 + 20)	-	33.0 (11.0), 36.0 (9.0)	0/20, 0/20	-	ASD	IQ 103.0 (20.0), 110.0 (13.0)	sMRI, 3T, slice thickness = 1.1mm	Sens and Spec
Neeley 2007	Research article	Training, internal	Others (university and community)	57 (= 33 +24)	-	13.9 (6.0), 13.3 (5.2)	0/33, 0/24	-	ASD	PIQ 97.1 (20.0), 100.2 (12.7)	sMRI, 3T	TP/FP/FN/NP

	Training, internal	Others (university and community)	57 (= 33 +24)	-	13.9 (6.0), 14.2 (6.2)	0/33, 0/24	-	ASD	PIQ 97.1 (20.0), 101.3 (12.3)	sMRI, 3T	TP/FP/FN/NP

Note: ABIDE, Autism Brain Imaging Data Exchange; NYU, New-York University (Langone Medical Center); KKI, Kennedy Krieger Institute; ASD, autism spectrum disorder; sMRI, structural MRI; Rs-fMRI, Resting state-functional Magnetic Resonance Imaging; Sens, Sensitivity; Sepc, Specificity; Acc, Accuracy; AUC, Area under the curve; Verbal ability, overall cognitive ability verbal ability, and non-verbal ability were measured by the Mullen Scales of Early Learning; PPV, Positive Predictive Value; NPV, Negative Predictive Value; USM, University of Utah School of Medicine; BNI, Barrow Neurological Institute; UM, University of Michigan; UCLA, University of California Los Angeles; LEUVEN, Katholieke Universiteit Leuven; IQ, (Mean full scale of) Intelligence Quotient; AD, Autistic Disorder; TP/FP/FN/NP, Ture-positive/False-positive/False-negative/Ture-negative; EEG, Electroencephalography; NDAR, National Database for Autism Research; DAS-II, Differential Ability Scales-II; PDD-NOS, Pervasive Developmental Disorder-Not Otherwise Specified; LC-MS/MS, liquid chromatography-tandem mass spectrometry; LR, Likelihood Ratio; UAR, Unweighted Average Recall; PEP-3, Psychoeducational Profile-3; DQ, Developmental Quotient; Mcc, Matthews correlation coefficient; FPR, False Positive Rate; FNR, False Negative Rate; ASC, Autism Spectrum Condition; WAIS, Wechsler Adult Intelligence Scale; MSEL, Mullen Scales of Early Learning; IBIS, Infant Brain Imaging Study; Pitt, University of Pittsburgh; ADDM, Autism and Developmental Disabilities Monitoring; NVIQ, IQ was measured by the Combined Raven Test; fNIRS, Functional near-infrared spectroscopy; RSPM, Raven's Standard Progressive Matrices; AC, Boston Autism Consortium; AGRE, autism genetic resource exchange; SSC, Simons Simplex Collection; IBR, Institute for Basic Research; BAR, Behavioral Assessment and Research; INFANT, IBR Infant Development Laboratory; Q- GLO, Queen's Genomics Lab at Ongwanada; ICD, Institute for Child Development; LR, Likelihood Ratio; LC-HRMS; Liquid Chromatography-High Resolution Mass Spectrometry; NDAR, National Database for Autism Research; WISC, Wechsler Intelligence Scale for Children; PIQ, performance IQ;
