# Supplementary material for: Accuracy of Machine Learning Algorithms for the Diagnosis of Autism Spectrum Disorder: Systematic Review and Meta-Analysis of Brain Magnetic Resonance Imaging Studies
Source: JMIR Ment Health. 2019 Dec 20;6(12):e14108. doi: 10.2196/14108 (PMC6942187; doi:10.2196/14108)
Supplement: Multimedia Appendix 6 [file mental_v6i12e14108_app6.pdf]

Multimedia Appendix 6. Characteristics of the performance and validation condition (details).

	Diagnostic workflow of algorithm						Reference standard of validation dataset			
	Acquisition/Pre-processing	Segmentation	Extracted (learning) features	Method of feature selection/extraction	Classification		Validation subtype	Diagnosis criteria of autism cases	Definition of autism +/-	Other diagnosis tool
					Specific algorithm	Predictors				
Kong 2018	FreeSurfer (motion correction, intensity normalization, skull stripping and cerebellum removal)	Destrieux atlas (148 cortical regions)	Gray matter volume (GMV), cortical thickness (CT) and standard deviation of cortical thickness (CTstd)	Filter (ranking)	DNN (with autoencoder)	Structural MRI features	Internal validation	DSM-Ⅳ-TR	ASD/TD	ADOS, ADI-R and review history
							External validation	-	-	-
Wan 2018	N/A	AOI of eye tracker fields (10 regions) by the unknown method	AOI fixation time	Others (direct extraction)	SVM	Behavior trait (eye fixation time)	Internal validation	DSM-5	ASD/TD	ADOS, ADI-R and CARS
Shen 2018	Distortion-corrected, mutual registration, transformation to stereotactic space	FSL Extraction Tool (majority voting approach), AutoSeg pipeline	Total cerebral volume (TCV) and extra-axial CSF	Others (direct extraction)	Ensemble (balance-boosted trees ensemble algorithm using RUSBoost trees)	Structural MRI features	External validation	ADOS and ADI-R	ASD/TD	-
Sharma 2018	N/A	N/A	Knowledge elicitation phase (description of symptoms)	Manually (human input)	Fuzzy (hierarchical)	Behavior trait	Internal validation	DSM-Ⅳ	ASD/TD	ADI-R
Mastrovito 2018	FSL (motion correction, linear registration)	Infomap algorithm (283 regions)	Functional relationships probabilities between areas	Wrapper (recursive feature elimination)	SVM	Functional MRI features	Internal validation	-	ASD/DD (schizophrenia only)	-
							External validation	ADOS-2	ASD/Controls	-
Li 2018	Connectome Computation System	Automated Anatomical Labeling atlas	Functional connectome patterns	Filter (weighting)	DNN (with autoencoder, transfer learning)	Functional MRI features	Internal validation	-	ASD/Controls	-
Heunis 2018	FIR filter (1–70 Hz), EEGLAB for rejecting artefact-ridden epochs	N/A	Recurrence event of EEG	Others (PCA, RQA etc.)	DA (linear), ANN (MLP), SVM	EEG features only or EEG features + sociodemographic features	Internal validation	DSM-Ⅳ-TR	ASD/TD	ADOS
Heinsfeld 2018	C-PAC (time and motion correction etc.), CompCor (Nuisance signal removal)	CC200 functional parcellation atlas (200 regions)	Functional connectivity (index of the level of co-activation of brain regions based on the time-series)	Filter (correlation)	SVM, RF or DNN (with autoencorder)	Functional MRI features	Internal validation	ADOS	ASD/TD	-
Dekhil 2018	BET (skull stripping), MCFLIRT (motion correction), SNR, FWHM	MNI152 standard Space (152 regions)	Functional connectivity (minimal loss decomposition of the source signal into two independent components)	Others (probabilistic independent component analysis etc.)	SVM (with autoencorder)	Functional MRI features	Internal validation	ADOS	ASD/TD	-
Castelhano 2018	Acquire Data Acquisition (ver 4.3.1), low-pass filter of 300 Hz	N/A	Time-frequency of EEG	Filter (correlation)	SVM	EEG features only	Internal validation	DSM-5	ASD/Controls	ADOS and ADI-R
Bernas 2018	FSL (motion correction, non-brain tissue removal etc.), FWHM	Socio-executive resting-state networks (by independent	Functional connectivity (wavelet coherence maps; resting-state networks their associated time series)	Others (mixed of extraction and selection)	DA (linear), SVM (with polynomial or Radial Basis Function)	Functional MRI features	External validation	DSM-IV-TR	ASD/TD	ADOS

		component analysis)								
Askari 2018	Selecting Epochs and removing artifacts by specific algorithm	N/A	Inter or intraregional connectivity (wavelet transform, variance, entropy)	Others (direct extraction)	SVM	EEG features only	Internal validation	DSM-IV-TR	ASD/ Controls	-
Anwar 2018	N/A	N/A	Plasma protein glycation, oxidation, and nitration adducts and amino acid metabolome in plasma and urine	Others (direct extraction)	SVM	Biochemical features from blood or urine	Internal validation	DSM-5	ASD/TD	ADOS and CARS
Abbas 2018	N/A	N/A	Description of behavioral patterns	Others (direct extraction by questionnaire)	Ensemble (decision trees)	Behavior trait and/or sociodemographic features	External validation	ADI-R and ADOS	Autism/ Controls	-
Xiao 2017	FreeSurfer, automated skull-stripping	Desikan–Killiany Cortical Atlas (66 regions)	Cortical volume (CV), cortical thickness (CT), regional area of cortical surfaces (CS)	Others (direct extraction)	RF, NB or SVM	Structural MRI features only	Internal validation	DSM-IV	PDD/DD	ADI-R and ADOS
Nakai 2017	Signal Processing Toolbox (software)	Sound editing software (spwave)	Fundamental frequency of voice (single-word utterance)	Filter (regression)	SVM	Voice (utterance)	Internal validation	DSM-5	ASD/TD	ADI-R
Oh 2017	Background correction and normalization by specific software	N/A	Gene expressed data (differentially expressed probes)	Filter (correlation)	HC, SVM, KNN or DA (linear)	Biochemical features from gene	Internal validation	DSM-IV-TR	ASD/TD	-
Hazlett 2017	Correction of intensity and distortions by specific algorithm	Automated, Anatomical Labeling (AAL) atlas (78 regions)	Cortical thickness and surface area measurements	Others (dimensional reduction and embedded)	SVM	Structural MRI features	Internal validation	DSM-IV	ASD/TD	ADOS
Emerson 2017	Sinc interpolation, interleaved acquisition, and spatial realignment	Adopted from a combination of meta-analyses of ASD studies (230 regions)	Functional connectivity	Others (correlation and dimension reduction)	SVM	Functional MRI features	Internal validation	DSM-IV-TR	ASD/TD	ADOS
Chaddad 2017	-	Labeled (segmented) manually by radiologist	Texture features (GLCM)	Filter (correlation)	SVM	Structural MRI features only	Internal validation	ADI-R and ADOS	ASD/TD	-
Bosl 2017	NetStation software (exclude segments with eye saccades)	N/A	Recurrence rate, determinism, laminarity, max line length, entropy and trapping time	Embedded	SVM	EEG features only	Internal validation	ADOS	ASD/Controls	-
Maenner 2016	N/A	N/A	Relative frequency of a word or phrase in evaluations	Embedded	RF	Text (words or phrase)	-	Surveillance ASD case definition	ASD (PDDNOS or AD)/ Controls	ADDM ASD criteria
Liu 2016	N/A	AOI of eye tracker fields (10 regions) by K-means clustering	Frequency or its difference of fixation time	Others (transformed to histogram)	SVM	Behavior trait (eye fixation time)	Internal validation	DSM-IV	ASD/TD	AQ-Child (Chinese)
Li 2016	N/A	N/A	Functional connectivity (temporal correlation coefficient of HbO2 and Hb signal)	Others (filter and dimensional reduction)	SVM	Biochemical features (concentration changes of HbO2 and Hb of blood)	Internal validation	DSM-IV-TR	ASD/TD	-

Duda 2016	N/A	N/A	Items of survey (SRS)	Others (ranking and forward feature selection)	DT, RF, SVM, DA (linear), regression (logistic) or Lasso	Behavior traits (identified by survey question)	Internal validation	-	ASD/DD (ADHD only)	-
Cohen 2016	N/A	N/A	Domain T-scores of survey (PDDBI)	Filter (intercorrelation)	DT (Classification and Regression Trees, CART)	Behavior traits (identified by survey question)	Internal validation	DSM-IV, DSM-IV-TR	ASD/Controls (DD + TD)	ADOS, ADI
Bone 2016	N/A	N/A	Items of survey (ADI-R and SRS)	Wrapper (greedy forward-feature selection)	SVM	Behavior traits (identified by survey question) and/or sociodemographic features	Internal validation	Diagnostic algorithm	ASD/Controls (DD + TD)	ADI-R, SRS
Promparo 2015	Array processing and gene filtering	Gene dendrogram and module colors	Leukocyte RNA coexpression (module eigengene)	Embedded	Regression (logistic)	Biochemical features from gene	Internal validation	DSM-IV-TR	ASD/Controls (DD + TD)	ADOS
Katuwal 2015	FreeSurfer (Recon-all workflow for motion correction)	FreeSurfer (Recon-all workflow for segmentation of cortical and subcortical structures)	Sematic (surface area, volume, thickness) and agnostic (Gaussian and mean curvature, folding index, thickness standard deviation) features	Various (wrapper, embedded or direct extraction)	RF, SVM or Gradient Boosting Machine (GBM)	Structural MRI features	Internal validation	DSM-IV-TR	ASD/TD	ADI-R, ADOS
Lidaka 2015	SPM8 and DPARSF (normalization to MNI, head motion correction and noise reducing)	Automated, Anatomical Labeling (AAL) atlas (90 regions)	Functional connectivity (effect size matrix of the difference between groups)	Filter (correlation)	PNN	Functional MRI features	Internal validation	DSM-IV-TR	ASD/TD	ADI-R, ADOS
Crippa 2015	5th-order Butterworth, 8-Hz low-pass filter	Segmentation was computed with self-written software	Three-dimensional kinematic data (total movement duration, number of movement units or peak velocity etc.)	Filter (ranking)	SVM	Behavior trait	Internal validation	DSM-IV-TR	ASD/TD	ADOS
West 2014	Filters (removing low abundance levels, defects)	Obiwarp algorithm (based on a non-linear clustering approach)	Molecular mass features (mass-to-charge ratio (m/z) and the chromatographic retention time)	Embedded	DA (PLS), SVM	Biochemical features	External validation	DSM-IV	AD/TD	ADOS-G, ADI-R
Wee 2014	-	Desikan-Killiany (68 regions) and Subcortical Structure (37 regions) Atlas	Regional (cortical thickness, GM and cortical associated WM, and subcortical structure volumes) and interregional morphological patterns (similarity map) from sMRI	Others (filter and wrapper combined)	SVM	Structural MRI features only	Internal validation	ADOS and ADI-R	ASD/Controls	-
Price 2014	fMRI DPARSF (normalization to MNI, head motion regression, motion scrubbing, etc.)	MELODIC software (25 statistically independent spatial maps)	Functional connectivity (intra-network, voxel-wise mean and variance of windowed correlation)	Filters (post-processing algorithm using cutoff, correlation test, and logistic regression)	SVM (using single or multi-kernel)	Functional MRI features	Internal validation	DSM-IV-TR	ASD/TD	ADI-R, ADOS
Uddin 2013	SPM (removing motion artifacts)	MELODIC software (25 statistically independent spatial maps)	Functional connectivity (intrinsic functional connectivity of brain networks)	Others (independent component analysis, dual-regression and clustering)	Regression (logistic)	Functional MRI features	Internal and external validation	ADOS and ADI-R	ASD/TD	-

Wang 2012	-	Automated, Anatomical Labeling (AAL) atlas (106 regions)	Functional connectivity (correlation corresponding to the resting and task trials)	Others (connectivity clustering geometry)	Regression (logistic)	Functional MRI features	Internal validation	ADOS	ASD/TD	-
Wall 2012 (1)	N/A	N/A	Items of survey (ADOS)	Embedded	DT (ADTree, BFTree, CART or Decision Stump etc.)	Behavior trait	Internal validation	Diagnostic algorithm	Autism/non-autism	ADOS
Wall (2) 2012	N/A	N/A	Items of survey (ADI-R)	Embedded	DT (ADTree, BFTree, CART or Decision Stump etc.)	Behavior trait	Internal or external validation	Diagnostic algorithm	Autism/non-autism	ADOS, ADI-R,
Calderoni 2012	VBM and DARTEL algorithm (detecting anatomical abnormalities etc.)	SPM default segmenting of brain tissues	Gray matter (GM), white matter (WM), cerebrospinal fluid (CSF) absolute volumes and total intracranial volume (TIV)	Embedded (SVM recursive feature elimination)	SVM	Structural MRI features	Internal validation	DSM-IV-TR	Autism/ Controls (DD + TD)	ADOS-G, CARS
Jiao 2010	FreeSurfer (gray and white boundary, perform automated topology correction etc.)	FreeSurfer (cortical reconstruction and volumetric segmentation)	Average cortical thickness or volume based measurements	Others (direct extraction)	SVM, MLP, DT (functional trees or logistic model trees)	Structural MRI features	Internal validation	DSM-IV	ASD/TD	ADI-R, CARS
Ecker 2010[a]	SPM5	Automated Talairach and Tournoux segmented	Gray matter (GM), white matter (WM) and cerebrospinal fluid (CSF) volume	Embedded (SVM recursive feature elimination)	SVM	Structural MRI features	Internal validation	ICD-10	ASD/TD	ADI-R, ADOS
Ecker 2010[b]	Freesurfer	Automated Talairach transformation and segmentation	Convexity or concavity, mean (radial) curvature, and metric distortion accounted for geometric features at each cerebral vertex and cortical thickness and surface area measured volumetric features	Others (direct extraction)	SVM	Structural MRI features	Internal validation	ICD-10	ASD/Controls	ADI-R, ADOS
Neeley 2007	-	ANALYZE multispectral tool	Quantification of temporal lobe structures (white matter, gray matter, and cerebral spinal fluid pixels and volumes)	Others (Discriminant analysis)	DT (CART)	Structural MRI features	Internal validation	DSM-IV	ASD/TD or ASD/DD (reading delay)	ADI-R, ADOS-G

Note: DNN, Deep Neural Network; DSM, Diagnostic and Statistical Manual of Mental Disorders; ASD, Autism Spectrum Disorder; TD, Typically Developing; ADOS, Autism Diagnostic Observation Schedule; ADI-R, Autism Diagnostic Interview-Revised; AOI, Areas of Interest; SVM, Support Vector Machine; FOCM/TS, Folate-dependent One Carbon Metabolism and Trans-Sulfuration pathways; DA, Discriminant Analysis; FSL, FMRIB Software Library; PCA, Principal Component Analysis; AD, Autistic Disorder; FIR, Finite Impulse Response; RQA, Recurrence Quantification Analysis; ANN, Artificial Neural Network; MLP, Multilayer Perceptron; BET, Brain Extraction Tool; SNR, Signal to Noise Ration; FWHM, Full Width Half Maximum; MNI152, Montreal Neurological Institute 152; NB, Naïve bayes; DD, Development Delay; PDD, Pervasive Developmental Disorders; DT, Decision Tree; HC, Hierarchical Cluster; KNN, K-nearest Neighbors; GLCM, Grey Level Co-occurrence Matrix; AQ, Autism Spectrum Quotient; SRS, Social Responsiveness Scale; PDDBI, PDD Behavior Inventory; RNA, RiboNucleic Acid; DPARSF, Data Processing Assistant for Resting-State fMRI; PNN, Probabilistic Neural Network; PLS, Partial Least Squares Discriminant Analysis; DPARSF, Data Processing Assistant for Resting-State; VBM, Voxel Based Morphometry; DARTEL, Diffeomorphic Anatomical Registration using Exponentiated Lie algebra; MLP, Multilayer Perceptron; ICD, International Statistical Classification of Diseases
