# Supplementary material for: Accuracy of Machine Learning Algorithms for the Diagnosis of Autism Spectrum Disorder: Systematic Review and Meta-Analysis of Brain Magnetic Resonance Imaging Studies
Source: JMIR Ment Health. 2019 Dec 20;6(12):e14108. doi: 10.2196/14108 (PMC6942187; doi:10.2196/14108)
Supplement: Multimedia Appendix 7 [file mental_v6i12e14108_app7.pdf]

Multimedia Appendix 7. QUADAS-2 assessment of all studies.

	Risk of bias (internal validity)				Applicability (external validity)		
	Patient selection	Index test	Reference standard	Flow and timing	Patient selection	Index test	Reference standard
Kong 2018	unclear	low	Low	low	low	low	low
Wan 2018	unclear	unclear	Low	low	low	low	low
Shen 2018	unclear	low	Low	low	low	low	low
Sharma 2018	unclear	high	unclear	low	low	high	low
Mastrovito 2018	unclear	low	unclear	unclear	high	low	low
Li 2018	unclear	unclear	low	unclear	unclear	low	unclear
Heunis 2018	unclear	unclear	low	low	low	low	low
Heinsfeld 2018	unclear	unclear	low	unclear	unclear	low	low
Dekhil 2018	unclear	unclear	low	low	low	low	unclear
Castelhano 2018	unclear	unclear	low	low	high	low	low
Bernas 2018	unclear	unclear	low	low	high	low	low
Askari 2018	low	unclear	unclear	low	low	low	low
Anwar 2018	low	unclear	low	high	low	low	low
Abbas 2018	low	low	low	low	unclear	low	low
Xiao 2017	unclear	unclear	low	low	high	low	low
Nakai 2017	unclear	unclear	low	low	low	low	low
Oh 2017	unclear	low	unclear	low	unclear	low	low
Hazlett 2017	low	unclear	low	low	unclear	low	low
Emerson 2017	low	unclear	low	low	high	low	low
Chaddad 2017	unclear	unclear	low	low	low	high	low
Bosl 2017	unclear	unclear	low	low	low	low	low
Maenner 2016	unclear	unclear	low	low	high	low	high
Liu 2016	unclear	unclear	low	low	low	low	low
Li 2016	unclear	low	low	low	low	low	low
Duda 2016	unclear	high	unclear	unclear	unclear	low	unclear
Cohen 2016	low	low	low	low	low	low	low

Bone 2016	unclear	unclear	low	unclear	low	low	high
Pramparo 2015	low	low	low	low	high	low	low
Katuwal 2015	unclear	unclear	low	low	unclear	low	low
Lidaka 2015	unclear	unclear	low	low	low	low	low
Crippa 2015	unclear	unclear	low	low	low	low	low
West 2014	unclear	low	low	low	low	low	low
Wee 2014	unclear	unclear	low	low	low	low	low
Price 2014	unclear	unclear	low	low	unclear	low	low
Uddin 2013	low	low	low	low	low	low	low
Wang 2012	unclear	unclear	unclear	low	unclear	low	low
Wall 2012 (1)	unclear	unclear	unclear	unclear	unclear	low	high
Wall 2012 (2)	unclear	low	unclear	unclear	unclear	low	high
Calderoni 2012	unclear	high	low	low	high	low	low
Jiao 2010	unclear	unclear	low	low	low	low	low
Ecker 2010 (1)	low	unclear	low	low	high	low	low
Ecker 2010 (2)	low	unclear	low	low	high	low	low
Neeley 2007	unclear	unclear	low	low	high	low	low
