# Supplementary material for: Accuracy of Machine Learning Algorithms for the Diagnosis of Autism Spectrum Disorder: Systematic Review and Meta-Analysis of Brain Magnetic Resonance Imaging Studies
Source: JMIR Ment Health. 2019 Dec 20;6(12):e14108. doi: 10.2196/14108 (PMC6942187; doi:10.2196/14108)
Supplement: Multimedia Appendix 8 [file mental_v6i12e14108_app8.pdf]

Multimedia Appendix 8. True positive (TP), false positive (FP), false negative (FN), true negative (TN), sensitivity and specificity of 53 study samples.

	Machine learning Type	Participations			Sensitivity (95% CI)	Specificity (95% CI)	TP	FP	FN	TN
		Total	ASD+	ASD-						
**Structural MRI subgroup (N = 12)**										
Shen 2018	SVM	236	159	77	0.83 (0.77-0.88)	0.65 (0.54-0.74)	133	27	26	50
Hazlett 2017	SVM	179	34	145	0.87 (0.72-0.95)	0.95 (0.90-0.97)	30	7	4	138
Chaddad 2017 (ABIDE-UM)	SVM	28	14	14	0.70 (0.45-0.87)	0.63 (0.39-0.83)	10	5	4	9
Chaddad 2017 (ABIDE-Pitt)	SVM	36	20	16	0.83 (0.63-0.94)	0.62 (0.39-0.81)	17	6	3	10
Wee 2014	SVM	117	58	59	0.94 (0.85-0.98)	0.96 (0.88-0.99)	55	2	3	57
Ecker 2010-1	SVM	44	22	22	0.85 (0.65-0.94)	0.85 (0.65-0.94)	19	3	3	19
Ecker 2010-2	SVM	40	20	20	0.88 (0.68-0.96)	0.88 (0.68-0.96)	18	2	2	18
Xiao 2017	RF	85	46	39	0.80 (0.66-0.89)	0.81 (0.67-0.90)	37	7	9	32
Katuwal 2015	RF	734	361	373	0.57 (0.52-0.62)	0.64 (0.59-0.69)	206	134	155	239
Jiao 2010	DT	38	22	16	0.89 (0.71-0.97)	0.74 (0.50-0.89)	20	4	2	12
Neeley 2007	DT	57	33	24	0.84 (0.68-0.93)	0.82 (0.63-0.92)	28	4	5	20
Kong 2018	DNN	182	78	104	0.84 (0.75-0.91)	0.96 (0.90-0.98)	66	4	12	100
Subtotal for structural MRI subgroup	4 types	1,776	867	909	0.57 to 0.94	0.62 to 0.96	639	205	228	704
**Functional MRI subgroup (N = 15)**										
Li 2018 (ABIDE-UM)	DNN	113	48	65	0.68 (0.54-0.80)	0.67 (0.55-0.78)	33	21	15	44
Li 2018 (ABIDE-UCLA)	DNN	75	36	39	0.55 (0.40-0.70)	0.69 (0.53-0.81)	20	12	16	27
Li 2018 (ABIDE-USM)	DNN	61	38	23	0.73 (0.58-0.84)	0.65 (0.45-0.80)	28	8	10	15
Li 2018 (ABIDE-LEUVEN)	DNN	61	27	34	0.66 (0.48-0.81)	0.70 (0.54-0.83)	18	10	9	24
Heinsfeld 2018	DNN	1,035	505	530	0.74 (0.70-0.78)	0.63 (0.59-0.67)	374	196	131	334
Dekhil 2018	SVM	283	112	171	0.90 (0.83-0.94)	0.88 (0.82-0.92)	101	21	11	150
Bernas 2018 (ABIDE-LEUVEN)	SVM	30	12	18	0.81 (0.54-0.94)	0.87 (0.66-0.96)	10	2	2	16
Mastrovito 2018	SVM	54	27	27	0.73 (0.55-0.86)	0.88 (0.71-0.95)	20	3	7	24
Emerson 2017	SVM	59	13	46	0.82 (0.56-0.94)	0.99 (0.91-1.00)	11	0.5[a]	2	46
Price 2014	SVM	60	30	30	0.86 (0.69-0.94)	0.92 (0.77-0.98)	26	2	4	28
Uddin 2013 (clinic sample)	Regression	40	20	20	0.74 (0.53-0.88)	0.79 (0.57-0.91)	15	4	5	16
Uddin 2013 (NADR)	Regression	30	15	15	0.66 (0.42-0.84)	0.97 (0.76-1.00)	10	0.5[a]	5	15
Wang 2012	Regression	58	29	29	0.82 (0.65-0.92)	0.82 (0.65-0.92)	24	5	5	24
Lidaka 2015	PNN	640	312	328	0.92 (0.89-0.95)	0.88 (0.84-0.91)	288	38	24	290
Bernas 2018 (hospital sample)	DA	24	12	12	0.89 (0.62-0.97)	0.81 (0.54-0.94)	11	2	1	10
Subtotal for functional MRI subgroup	5 types	2,623	1,236	1,387	0.55 to 0.92	0.63 to 0.99	989	324	247	1,063
**Behavior trait subgroup (N = 14)**										
Cohen 2016 (randomly split-1)	DT	338	272	66	0.82 (0.77-0.86)	0.87 (0.77-0.93)	223	8	49	58

Cohen 2016 (randomly split-2)	DT	163	132	31	0.83 (0.76-0.86)	0.86 (0.70-0.94)	110	4	22	27
Cohen 2016 (randomly split-3)	DT	159	130	29	0.86 (0.79-0.91)	0.92 (0.77-0.97)	112	2	18	27
Wall 2012-1	DT	627	612	15	1.00 (1.00-1.00)	0.97 (0.76-1.00)	612	0.5[a]	0.5[a]	15
Wall 2012-2 (AGRE)	DT	966	891	75	1.00 (1.00-1.00)	0.98 (0.92-1.00)	891	1	0.5[a]	74
Wall 2012-2 (AC)	DT	1659	1,654	5	1.00 (1.00-1.00)	0.58 (0.24-0.86)	1,654	2	0.5[a]	3
Wall 2012-2 (SSC)	DT	344	332	12	0.97 (0.94-0.98)	0.50 (0.26-0.74)	321	6	11	6
Wan 2018	SVM	74	37	37	0.86 (0.71-0.93)	0.82 (0.68-0.92)	32	6	5	31
Liu 2016	SVM	87	29	58	0.92 (0.77-0.94)	0.86 (0.75-0.92)	27	8	2	50
Bone 2016 (age equal to above 10)	SVM	567	389	178	0.89 (0.86-0.92)	0.59 (0.52-0.66)	347	73	42	105
Bone 2016 (age below 10)	SVM	319	238	81	0.86 (0.82-0.90)	0.53 (0.42-0.64)	206	38	32	43
Crippa 2015	SVM	30	15	15	0.97 (0.76-1.00)	0.91 (0.68-0.98)	15	1	0.5[a]	14
Sharma 2018	Fuzzy	80	39	41	0.99 (0.89-1.00)	0.96 (0.86-1.00)	39	1	0.5[a]	40
Abbas 2018	Ensemble	162	121	41	0.68 (0.59-0.75)	0.56 (0.41-0.70)	82	18	39	23
Subtotal for behavior trait group	4 types	5,575	4,891	684	0.68 to 1.00	0.56 to 0.97	4,671	168.5	222.5	516
**Biochemical subgroup (N = 7)**										
Anwar 2018	SVM	48	27	21			24	3	3	18
Oh 2017	SVM	16	8	8	0.94 (0.63-0.99)	0.83 (0.51-0.96)	8	1	0.5[a]	7
Li 2016	SVM	24	13	11	0.82 (0.56-0.94)	0.88 (0.60-0.97)	11	1	2	10
West 2014 (randomly split-2)	SVM	21	13	8	0.86 (0.72-0.94)	0.93 (0.76-0.98)	12	3	1	5
Pramparo 2015 (randomly split-1)	Regression	142	87	55	0.85 (0.76-0.91)	0.79 (0.67-0.88)	74	11	13	44
Pramparo 2015 (randomly split-2)	Regression	73	44	29	0.77 (0.63-0.87)	0.72 (0.54-0.85)	34	8	10	21
West 2014 (randomly split-1)	DA	61	39	22	0.86 (0.72-0.94)	0.93 (0.76-0.98)	34	1	5	21
Subtotal for biochemical predictors group	3 types	385	231	154	0.77 to 0.94	0.72 to 0.93	197	28	34.5	126
**EEG subgroup (N= 3)**										
Heunis 2018	SVM	14	7	7	0.94 (0.60-0.99)	0.81 (0.47-0.96)	7	1	0.5[a]	6
Askari 2018	SVM	183	89	94	0.99 (0.95-1.00)	0.95 (0.89-0.98)	89	4	0.5[a]	90
Bosl 2017	SVM	41	18	23	0.97 (0.79-1.00)	0.94 (0.77-0.99)	18	1	0.5[a]	22
Subtotal for EEG group	1 type	238	114	124	0.94 to 0.97	0.81 to 0.94	114	6	1.5	118
**Voice features subgroup (N = 1)**										
Nakai 2017	SVM	81	30	51	0.69 (0.52-0.83)	0.80 (0.67-0.88)	21	10	9	41
**Text or word features subgroup (N = 1)**										
Maenner 2016	RF	1,450	754	696	0.97 (0.79-1.00)	0.89 (0.87-0.91)	633	75	121	621
**Overall (N = 53)**	**8 types**	**12,128**	**8,123**	**4,005**	**0.55 to 1.00**	**0.56 to 0.99**	**7,264**	**813**	**864**	**3,189**

Abbreviation: MRI, Magnetic Resonance Imaging; ABIDE, Autism Brain Imaging Data Exchange; NYU, New-York University (Langone Medical Center); USM,

University of Utah School of Medicine; UM, University of Michigan; UCLA, University of California Los Angeles; LEUVEN, Katholieke Universiteit Leuven; Pitt, University of Pittsburgh; NDAR, National Database for Autism Research; AC, Boston Autism Consortium; AGRE, autism genetic resource exchange; SSC, Simons Simplex Collection; DNN, Deep neural network; SVM, Support vector machine; RF, Random forest; DT, Decision tree; PNN, Probabilistic neural network; DA, Discriminate analysis; EEG, Electroencephalography.

a In order to prevent the problem of zero cell counts, we added 0.5 to the cells of the 2 × 2 contingency table if a value was 0 in TP/FP/TN/FN.
