# Supplementary material for: Accuracy of Machine Learning Algorithms for the Diagnosis of Autism Spectrum Disorder: Systematic Review and Meta-Analysis of Brain Magnetic Resonance Imaging Studies
Source: JMIR Ment Health. 2019 Dec 20;6(12):e14108. doi: 10.2196/14108 (PMC6942187; doi:10.2196/14108)
Supplement: Multimedia Appendix 9 [file mental_v6i12e14108_app9.pdf]

Multimedia Appendix 9. Pre-coded raw variable data sheet for subgroup analysis.

Total	D1S1 (Publication type)	D2S1 (Validation dataset-1)	D2S2 (Validation dataset-2)	D2S3 (Dataset type)	D2S4 (Age (6/18y))	D2S5 (Age (6y))	D2S6 (Age (18y))	D2S7 (Infant study)	D2S8 (Gender of validation dataset)	D2S9 (Matched)	D3S1 (Acquisition)	D3S2 (Segmentation)	D3S3 (Extraction)	D3S4 (Predictor)	D3S5 (Classifier techtree-1)	D3S6 (Classifer techtree-2)	D3S7 (Reference standard-2)	D3S8 (Autism character)	D3S9 (Control character)	D4S1 (쎄)	D4S2 (FP)	D4S3 (FN)	D4S4 (TN)
Kong 2018	Journal	Internal	Cross	Public	6 to 18	>6	<18	N	Mixed	Unknown	Y	Not_manually	Filter	Structural MRI	DNN	Others	DSM	ASD	TD	66	4	12	100
Wan 2018	Journal	Internal	Cross	Private	<6	<6	<18	N	Mixed	Y	NA	Unknown	Others	Behavior	SVM	Supervised	DSM	ASD	TD	32	6	5	31
Shen 2018	Journal	External	External	Private	<6	<6	<18	Y	Mixed	Unknown	Y	Not_manually	Others	Structural MRI	Ensemble	Supervised	Others	ASD	TD	133	27	26	50
Sharma 2018	Journal	Unknown	Unknown	Unknown	<6	<6	<18	N	Mixed	Y	NA	NA	Manually	Behavior	Fuzzy	Supervised	DSM	ASD	TD	39	1	0	40
Mastrovito 2018	Journal	External	External	Private	>18	>6	>18	N	Male only	Unknown	Y	Not_manually	Wrapper	Functional MRI	SVM	Supervised	Others	ASD	Control	20	3	7	24
Li 2018 (1)	Journal	Internal	Cross	Public	6 to 18	>6	<18	N	Mixed	Unknown	Y	Not_manually	Filter	Functional MRI	DNN	Others	Unknown	ASD	Control	33	21	15	44
Li 2018 (2)	Journal	Internal	Cross	Public	6 to 18	>6	<18	N	Mixed	Unknown	Y	Not_manually	Filter	Functional MRI	DNN	Others	Unknown	ASD	Control	20	12	16	27
Li 2018 (3)	Journal	Internal	Cross	Public	>18	>6	>18	N	Male only	Unknown	Y	Not_manually	Filter	Functional MRI	DNN	Others	Unknown	ASD	Control	28	8	10	15
Li 2018 (4)	Journal	Internal	Cross	Public	>18	>6	>18	N	Mixed	Unknown	Y	Not_manually	Filter	Functional MRI	DNN	Others	Unknown	ASD	Control	18	10	9	24
Heunis 2018	Journal	Internal	Cross	Private	<6	<6	<18	N	Mixed	Y	Y	NA	Others	EEG	SVM	Supervised	DSM	ASD	TD	7	1	0	6
Heinsfeld 2018	Journal	Internal	Cross	Public	6 to 18	>6	<18	N	Mixed	Y	Y	Not_manually	Filter	Functional MRI	DNN	Others	Others	ASD	TD	374	196	131	334
Dekhil 2018	Journal	Internal	Cross	Public	6 to 18	>6	<18	N	Mixed	Y	Y	Not_manually	Others	Functional MRI	SVM	Others	Others	ASD	TD	101	21	11	150
Bernas 2018 (1)	Journal	Internal	Cross	Private	6 to 18	>6	<18	N	Unknown	Y	Y	Not_manually	Others	Functional MRI	DA	Unsupervised	DSM	ASD	TD	11	2	1	10
Bernas 2018 (2)	Journal	Internal	Cross	Public	6 to 18	>6	<18	N	Unknown	Y	Y	Not_manually	Others	Functional MRI	SVM	Supervised	DSM	ASD	TD	10	2	2	16
Askari 2018	Journal	Internal	Cross	Private	6 to 18	>6	<18	N	Mixed	Unknown	Y	NA	Others	EEG	SVM	Supervised	DSM	ASD	Control	89	4	0	90
Anwar 2018	Journal	Internal	Cross	Private	6 to 18	>6	<18	N	Mixed	Y	NA	NA	Others	Biochem	SVM	Supervised	DSM	ASD	TD	24	3	3	18
Abbas 2018	Journal	External	External	Others	Unknown	Unknown	Unknown	N	Unknown	Unknown	NA	NA	Others	Behavior	Ensemble	Supervised	Others	Others (Autism)	Control	82	18	39	23
Xiao 2017	Journal	Internal	Cross	Private	<6	<6	<18	N	Mixed	Y	Y	Not_manually	Others	Structural MRI	RF	Supervised	DSM	PDD	Control	37	7	9	32
Nakai 2017	Journal	Internal	Cross	Private	6 to 18	>6	<18	N	Mixed	Unknown	Y	Not_manually	Filter	Voice	SVM	Supervised	DSM	ASD	TD	21	10	9	41
Oh 2017	Journal	Internal	Split	Public	Unknown	Unknown	Unknown	N	Unknown	Unknown	Y	NA	Filter	Biochem	SVM	Supervised	DSM	ASD	TD	8	1	0	7
Hazlett 2017	Others	Internal	Cross	Private	<6	<6	<18	Y	Unknown	Unknown	Y	Not_manually	Others	Structural MRI	SVM	Supervised	DSM	ASD	TD	30	7	4	138

Emerson 2017	Others	Internal	Cross	Private	<6	<6	<18	Y	Male only	Unknown	Y	Not_manually	Others	Functional MRI	SVM	Supervised	DSM	ASD	TD	11	0	2	46
Chaddad 2017 (1)	Journal	Internal	Cross	Public	6 to 18	>6	<18	N	Mixed	Y	N	Manually	Filter	Structural MRI	SVM	Supervised	Others	ASD	TD	10	5	4	9
Chaddad 2017 (2)	Journal	Internal	Cross	Public	6 to 18	>6	<18	N	Mixed	Y	N	Manually	Filter	Structural MRI	SVM	Supervised	Others	ASD	TD	17	6	3	10
Bosl 2017	Journal	Internal	Cross	Private	6 to 18	>6	<18	N	Mixed	Unknown	Y	NA	Filter	EEG	SVM	Supervised	Others	ASD	TD	18	1	0	22
Maenner 2016	Journal	Unknown	Unknown	Others	<6	<6	<18	N	Mixed	Unknown	NA	NA	Embeded	Text	RF	Supervised	Others	Others (PDDNOS + AD)	Control	633	75	121	621
Liu 2016	Journal	Internal	Cross	Private	6 to 18	>6	<18	N	Mixed	Y	N	Not_manually	Others	Behavior	SVM	Supervised	DSM	ASD	TD	27	8	2	50
Li 2016	Journal	Internal	Split	Private	6 to 18	>6	<18	N	Mixed	Y	NA	NA	Others	Biochem	SVM	Supervised	DSM	ASD	TD	11	1	2	10
Cohen 2016 (1)	Journal	Internal	Split	Others	<6	<6	<18	N	Mixed	Unknown	NA	NA	Filter	Behavior	DT	Supervised	DSM	ASD	Control	223	8	49	58
Cohen 2016 (2)	Journal	Internal	Split	Others	<6	<6	<18	N	Mixed	Unknown	NA	NA	Filter	Behavior	DT	Supervised	DSM	ASD	Control	110	4	22	27
Cohen 2016 (3)	Journal	Internal	Split	Others	<6	<6	<18	N	Mixed	Unknown	NA	NA	Filter	Behavior	DT	Supervised	DSM	ASD	Control	112	2	18	27
Bone 2016 (1)	Journal	Internal	Cross	Unknown	6 to 18	>6	<18	N	Mixed	Unknown	NA	NA	Wrapper	Behavior	SVM	Supervised	Others	ASD	Control	347	73	42	105
Bone 2016 (2)	Journal	Internal	Cross	Unknown	6 to 18	>6	<18	N	Mixed	Unknown	NA	NA	Wrapper	Behavior	SVM	Supervised	Others	ASD	Control	206	38	32	43
Prampparo 2015 (1)	Journal	Internal	Cross	Private	<6	<6	<18	Y	Male only	Unknown	Y	Not_manually	Embedded	Biochem	Regression	Supervised	DSM	ASD	Control	74	11	13	44
Prampparo 2015 (2)	Journal	Internal	Split	Private	<6	<6	<18	Y	Male only	Unknown	Y	Not_manually	Embedded	Biochem	Regression	Supervised	DSM	ASD	Control	34	8	10	21
Katuwal 2015	Journal	Internal	Cross	Public	Unknown	Unknown	Unknown	N	Unknown	Unknown	Y	Not_manually	Embedded	Structural MRI	RF	Supervised	DSM	ASD	TD	206	134	155	239
Lidaka 2015	Journal	Internal	Cross	Public	6 to 18	>6	<18	N	Mixed	Unknown	Y	Not_manually	Filter	Functional MRI	NN	Supervised	DSM	ASD	TD	288	38	24	290
Crippa 2015	Journal	Internal	Cross	Private	<6	<6	<18	N	Mixed	Y	Y	Not_manually	Filter	Behavior	SVM	Supervised	DSM	ASD	TD	15	1	0	14
West 2014 (1)	Journal	Internal	Cross	Private	<6	<6	<18	N	Mixed	Y	Y	Not_manually	Embedded	Biochem	DA	Unsupervised	DSM	Others (AD)	TD	34	1	5	21
West 2014 (2)	Journal	Internal	Split	Private	<6	<6	<18	N	Mixed	Y	Y	Not_manually	Embedded	Biochem	SVM	Supervised	DSM	Others (AD)	TD	12	3	1	5
Wee 2014	Journal	Internal	Cross	Public	6 to 18	>6	<18	N	Mixed	Y	N	Not_manually	Others	Structural MRI	SVM	Supervised	Others	ASD	Control	55	2	3	57
Price 2014	Others	Internal	Cross	Public	6 to 18	>6	<18	N	Unknown	Unknown	Y	Not_manually	Filter	Functional MRI	SVM	Supervised	DSM	ASD	TD	26	2	4	28
Uddin 2013 (1)	Journal	Internal	Cross	Private	6 to 18	>6	<18	N	Mixed	Y	Y	Not_manually	Others	Functional MRI	Regression	Supervised	Others	ASD	TD	15	4	5	16
Uddin 2013 (2)	Journal	External	External	Public	6 to 18	>6	<18	N	Mixed	Y	Y	Not_manually	Others	Functional MRI	Regression	Supervised	Others	ASD	TD	10	0	5	15
Wang 2012	Journal	Internal	Cross	Others	Unknown	Unknown	Unknown	N	Mixed	Unknown	N	Not_manually	Others	Functional MRI	Regression	Supervised	Others	ASD	TD	24	5	5	24

Wall 2012 (1-1)	Journal	Internal	Cross	Others	Unknown	Unknown	Unknown	N	Unknown	Unknown	NA	NA	Embedded	Behavior	DT	Supervised	Others	Others (Autism)	Control	612	0	0	15
Wall 2012 (2-1)	Journal	Internal	Cross	Others	6 to 18	>6	<18	N	Unknown	Y	NA	NA	Embedded	Behavior	DT	Supervised	Others	Others (Autism)	Control	891	1	0	74
Wall 2012 (2-2)	Journal	External	External	Others	6 to 18	>6	<18	N	Unknown	Y	NA	NA	Embedded	Behavior	DT	Supervised	Others	Others (Autism)	Control	1654	2	0	3
Wall 2012 (2-3)	Journal	External	External	Others	6 to 18	>6	<18	N	Unknown	Y	NA	NA	Embedded	Behavior	DT	Supervised	Others	Others (Autism)	Control	321	6	11	6
Jiao 2010	Journal	Internal	Cross	Private	6 to 18	>6	<18	N	Mixed	Y	Y	Not_manually	Others	Structural MRI	DT	Supervised	DSM	ASD	TD	20	4	2	12
Ecker 2010 (1)	Journal	Internal	Cross	Others	>18	>6	>18	N	Male only	Y	Y	Not_manually	Embedded	Structural MRI	SVM	Supervised	Others	ASD	TD	19	3	3	19
Ecker 2010 (2)	Journal	Internal	Cross	Others	>18	>6	>18	N	Male only	Y	Y	Not_manually	Others	Structural MRI	SVM	Supervised	Others	ASD	TD	18	2	2	18
Neeley 2007	Journal	Unknown	Unknown	Others	6 to 18	>6	<18	N	Male only	Y	N	Not_manually	Others	Structural MRI	DT	Supervised	DSM	ASD	TD	28	4	5	20
