# Supplementary figures and images for: Accuracy of Machine Learning Algorithms for the Diagnosis of Autism Spectrum Disorder: Systematic Review and Meta-Analysis of Brain Magnetic Resonance Imaging Studies
Source: JMIR Ment Health. 2019 Dec 20;6(12):e14108. doi: 10.2196/14108 (PMC6942187; doi:10.2196/14108)

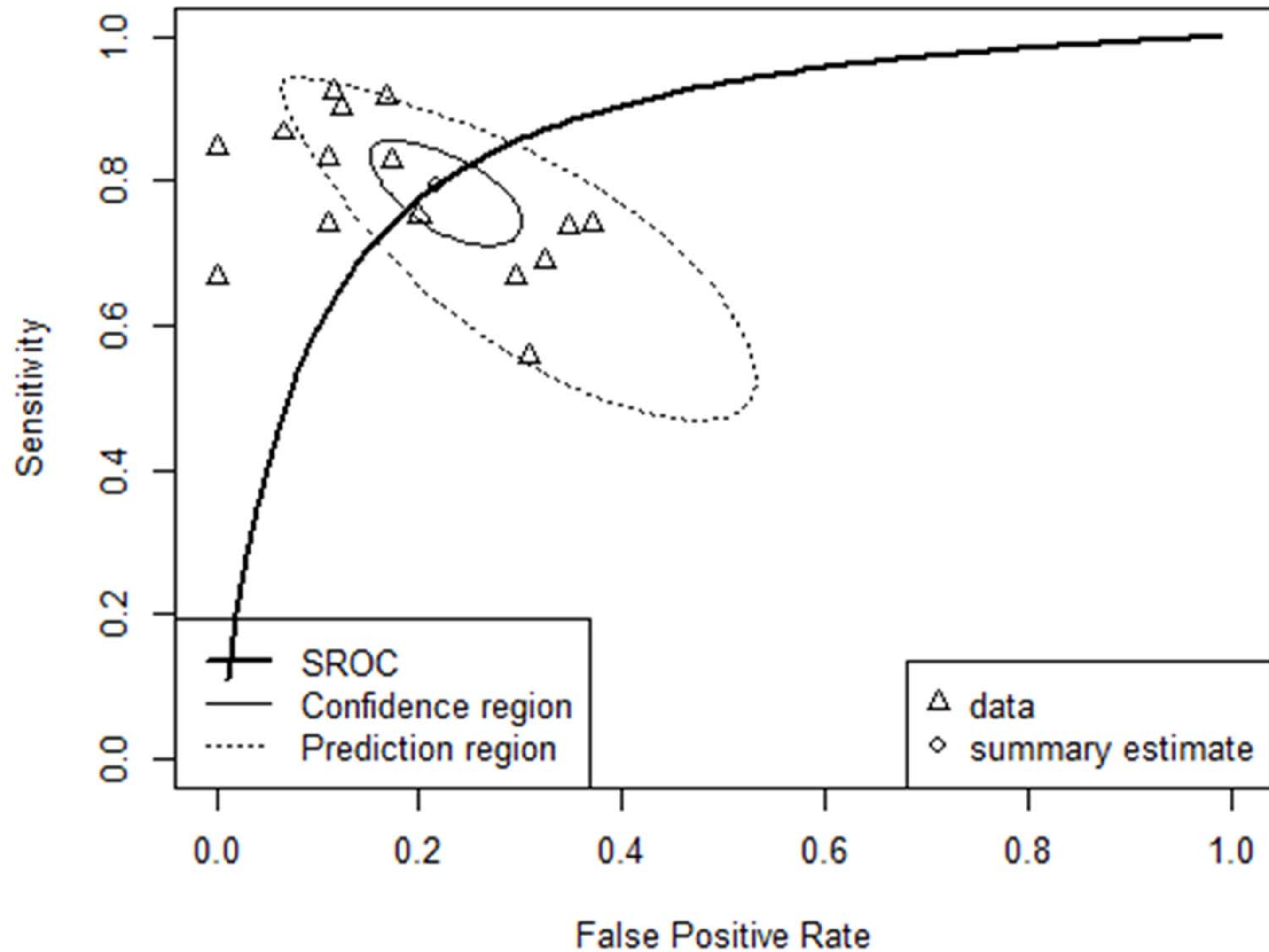

Supplement: Multimedia Appendix 10 [file mental_v6i12e14108_app10.pdf]

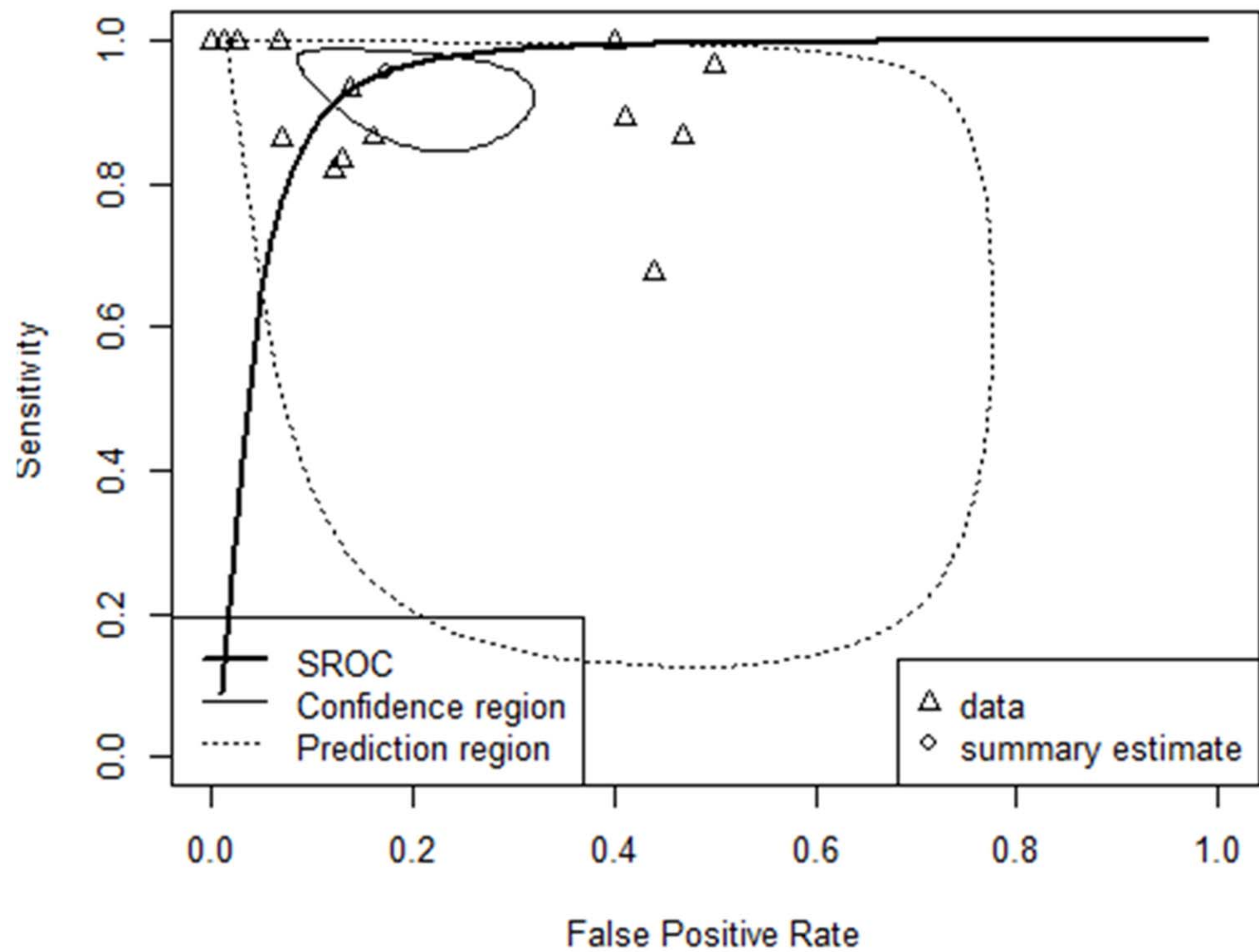

Supplement: Multimedia Appendix 11 [file mental_v6i12e14108_app11.pdf]

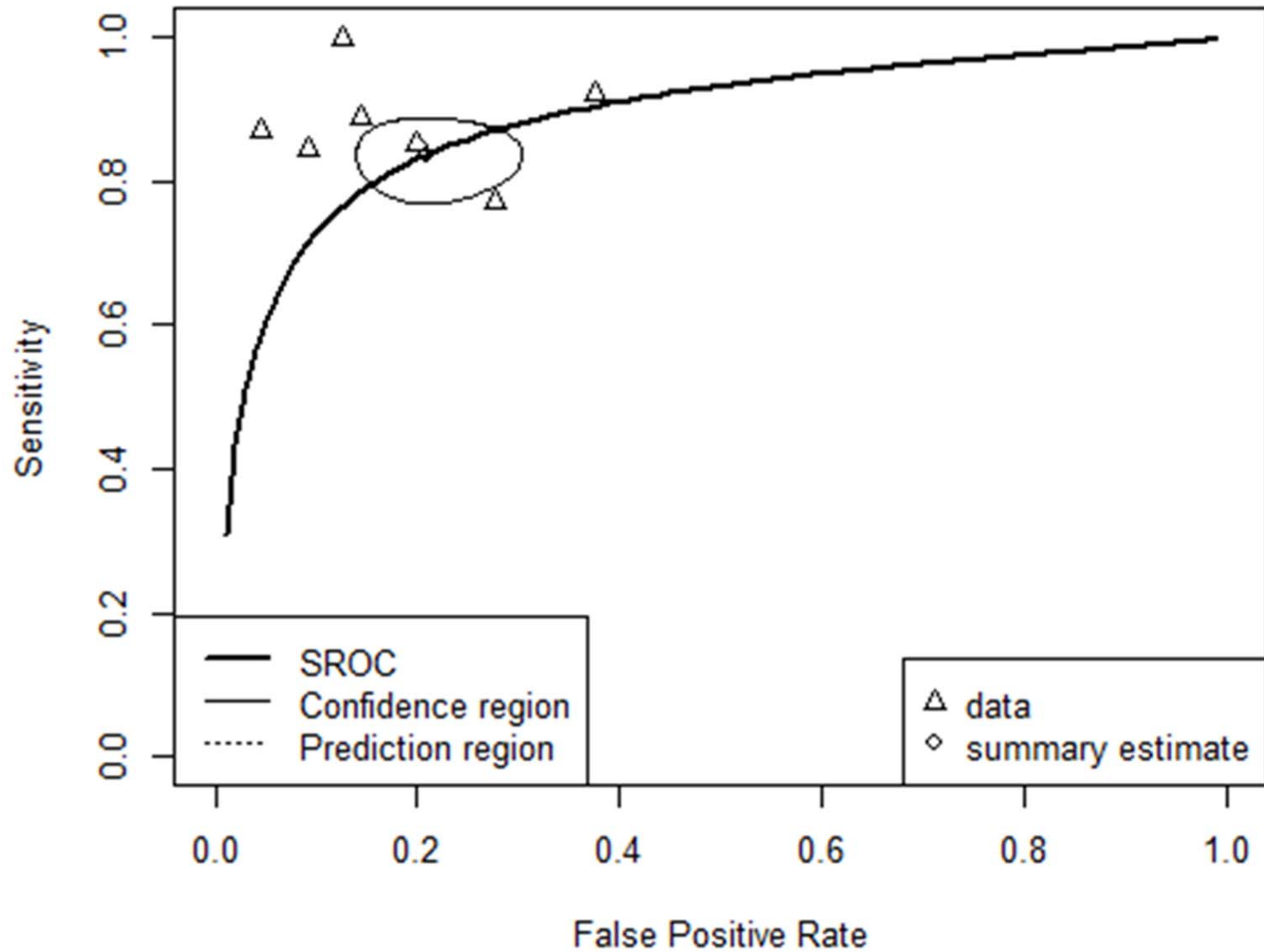

Supplement: Multimedia Appendix 12 [file mental_v6i12e14108_app12.pdf]

## Multimedia Appendix 13. Forest plot of structural and functional MRI subgroup..

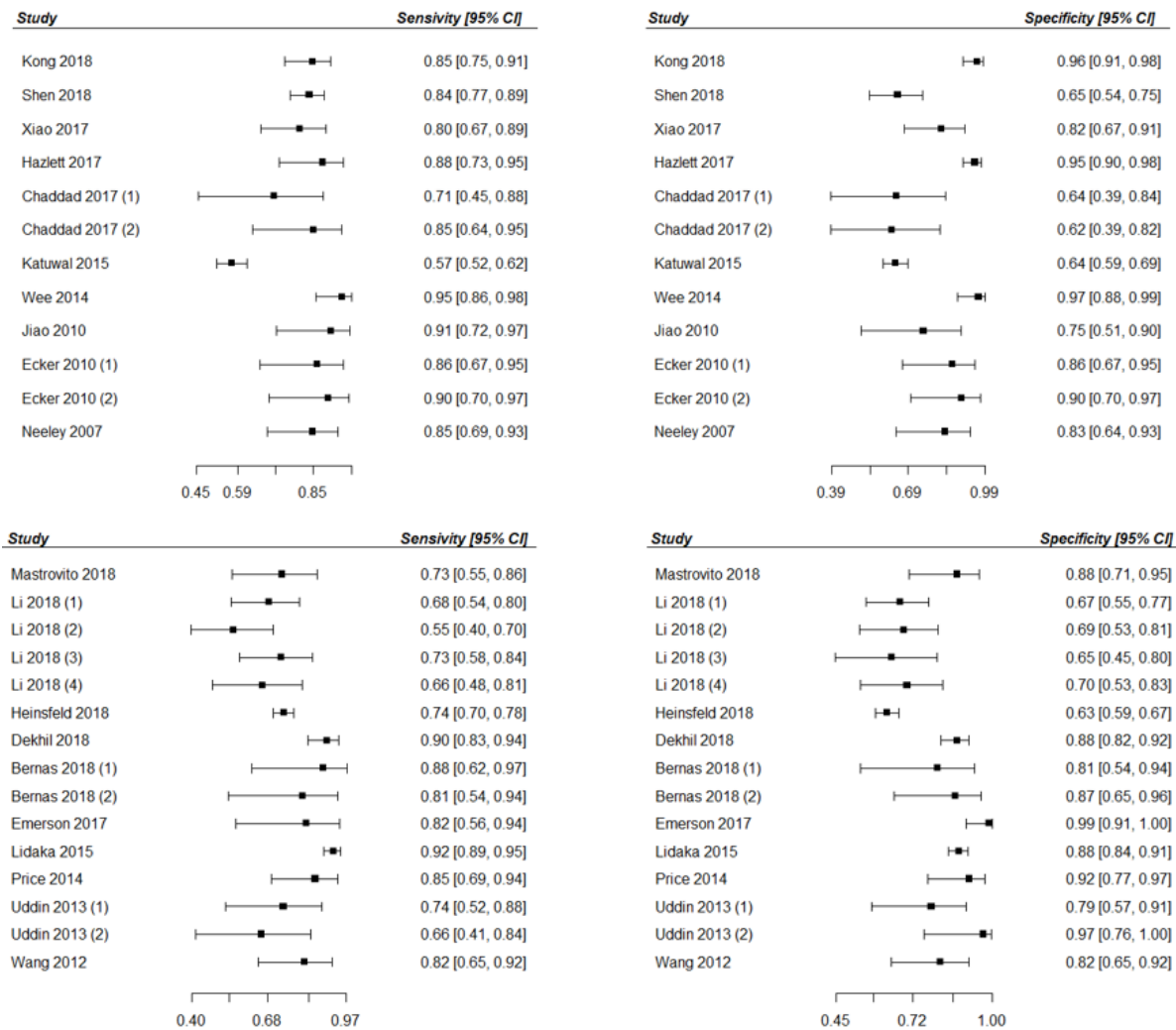

Supplement: Multimedia Appendix 13 [file mental_v6i12e14108_app13.pdf]
