# Supplementary material for: Accuracy of Machine Learning Algorithms for the Diagnosis of Autism Spectrum Disorder: Systematic Review and Meta-Analysis of Brain Magnetic Resonance Imaging Studies
Source: JMIR Ment Health. 2019 Dec 20;6(12):e14108. doi: 10.2196/14108 (PMC6942187; doi:10.2196/14108)
Supplement: Multimedia Appendix 14 [file mental_v6i12e14108_app14.pdf]

Multimedia Appendix 14. Excluded Accuracy indices for meta-analysis.

Author, year	Validation dataset	Validation method	Reference standard	Predictor type	Type of extractor	Type of classifier	Sample size for validation	AUC	Other indices (reported)
Kong 2018	Public	Internal (cross)	DSM-Ⅳ-TR	Structural MRI	Filter	DNN	182	0.97	Acc = 0.90
	Public	External					-	0.98	Acc = 0.87
Wan 2018	Private	Internal (cross)	DSM-5	Behavior traits	Others	SVM	74	-	Acc = 0.85
Shen 2018	Private	External	ADOS and ADI-R	Structural MRI	Others	Ensemble	236	-	Acc = 0.78
Sharma 2018	-	Internal	DSM-Ⅳ	Behavior traits	Manually	Fuzzy	80	-	Acc = 0.99
Mastrovito 2018	Mixed	Internal	-	Functional MRI	Wrapper	SVM	-	-	Acc = 0.75
	Public	Internal (cross)	-	Functional MRI	Wrapper	SVM	54	-	Acc = 0.83
Li 2018	Public	Internal (cross)	-	Functional MRI	Filter	DNN	113	0.67	Acc = 0.67
	Public	Internal (cross)	-	Functional MRI	Filter	DNN	75	0.69	Acc = 0.62
	Public	Internal (cross)	-	Functional MRI	Filter	DNN	61	0.73	Acc = 0.70
	Public	Internal (cross)	-	Functional MRI	Filter	DNN	61	0.74	Acc = 0.68
Heunis 2018	Private	Internal (cross)	DSM-Ⅳ-TR	EEG features only	Others	SVM	62	-	Acc = 0.87
				EEG features + sociodemographic features			62	-	Acc = 0.85 to 0.97
	Private (age matched subsample)	Internal (cross)	DSM-Ⅳ-TR	EEG features only	Others	SVM	14	-	Acc = 0.94
				EEG features + sociodemographic features			14	-	Acc = 0.91 to 0.96
Heinsfeld 2018	Public	Internal (cross)	ADOS	Functional MRI	Filter	SVM	1,035	-	Acc/Sens/Spec = 0.65/0.68/0.62
						RF	1,035	-	Acc/Sens/Spec = 0.63/0.69/0.58
Dekhil 2018	Public	Internal (cross)	ADOS	Functional MRI	Others	SVM	-	0.93	Acc/Sens/Spec = 0.92/0.93/0.89
Castelhano 2018	Private	Internal (cross)	DSM-5	EEG features only	Filter	SVM	20	0.99	Acc = 0.85 to 0.99
Bernas 2018	Private	Internal (cross)	DSM-Ⅳ-TR	Functional MRI	Others	LDA	24	-	Acc/Sens/Spec = 0.75, 0.88/0.83, 0.92/0.67, 0.83
						SVM	24	-	Acc/Sens/Spec = 0.71 to 0.87/0.67 to

									0.83/0.58 to 0.83
	Public	Internal (cross)	DSM-Ⅳ-TR	Functional MRI	Others	LDA	30	-	Acc/Sens/Spec = 0.57 to 0.83/0.67 to 1.00/0.28 to 0.83
						SVM	30	-	Acc/Sens/Spec = 0.60 to 0.87/0.75 to 1.00/0.39 to 0.83
Askari 2018	Private	Internal (cross)	DSM-Ⅳ-TR	EEG features only	Others	SVM	183	-	Acc = 0.95 to 1.00
Anwar 2018	Private	Internal (mixed)	DSM-5	Biochemical features	Others	SVM	50% of 69	0.79 to 0.95	Acc/Sens/Spec = 0.75 to 0.89/0.77 to 0.92/0.67 to 0.87
Abbas 2018	Private	External	ADI-R and ADOS	Behavior traits	Others	Ensemble	162	0.62 to 0.69	Sens/Spec = 0.64 to 0.68/0.48 to 0.57
Xiao 2017	Private	Internal (cross)	DSM-Ⅳ	Structural MRI	Others	RF	85	0.88	Acc/Mcc = 0.81/0.62
						NB	85	0.79	Acc/Sens/Spec/Mcc = 0.80/0.86/0.73/0.59
						SVM	85	0.79	Acc/Sens/Spec/Mcc = 0.80/0.85/0.74/0.59
Nakai 2017	Private	Internal (cross)	DSM-5	Voice features	Filter	SVM	81	-	Acc/Sens/Spec = 0.45 to 0.77/0.47 to 0.80/0.33 to 0.93
Oh 2017	Public	Internal (split)	DSM-Ⅳ-TR	Biochemical features	Filter	SVM	16	-	Acc = 0.94
						KNN	16	-	Acc/Sens/Spec = 0.94/1.00/0.89
						DA	16	-	Acc/Sens/Spec = 0.69/0.63/0.75
		Internal (cross)	DSM-Ⅳ	Biochemical features	Filter	HC	42	0.94	Sens = 0.86
Hazlett 2017	Private	Internal (cross)	DSM-Ⅳ	Structural MRI	Others	SVM	179	-	Acc = 0.93
Emerson 2017	Private	Internal (cross)	DSM-Ⅳ-TR	Functional MRI	Others	SVM	59	-	Acc = 0.97
Chaddad 2017	Public	Internal (cross)	ADI-R and ADOS	Structural MRI	Filter	SVM	28	-	Acc = 0.52 and 0.68
							36	-	Acc = 0.50 and 0.75
Bosl 2017	Private	Internal (cross)	ADOS	EEG features only	Filter	SVM	41	-	Acc = 0.97

Maenner 2016	Others	-	Surveillance ASD case definition	Text (words or phrase)	Embedded	RF	1,450	0.93	-
Liu 2016	Private	Internal (cross)	DSM-IV	Behavior traits	Others	SVM	-	0.90	Acc = 0.89
Li 2016	Private	Internal (split)	DSM-IV-TR	Biochemical features	Others	SVM	24	-	PPV = 0.88
Duda 2016	Others	Internal (cross)	-	Behavior traits	Others	DT	-	0.933	-
						RF	-	0.952	-
						SVC	-	0.965	-
						Regression (logistic)	-	0.962	-
						Lasso	-	0.962	-
						DA (linear)	-	0.964	-
Cohen 2016	Others	Internal (split into 3 groups)	DSM-IV	Behavior traits	Filter	DT	338/163/159	-	PPV = 0.97/0.96/0.98, NPV = 0.54/0.55/0.60, LR+/- = 4.58/0.20
Bone 2016	-	Internal (cross)	Algorithmic diagnosis	Behavior traits and/or sociodemographic features	Wrapper	SVM	567/319	-	UAR = 0.80 for age 10 – group, 0.77 for age 10 + group
Pramparo 2015	Private	Internal (cross)	DSM-IV-TR	Biochemical features	Embedded	Regression (logistic)	142	0.87	Acc = 0.83
		Internal (split)					73	0.77	Acc = 0.75
Katuwal 2015	Public	Internal (cross)	DSM-IV-TR	Structural MRI	Embedded	RF/SVM/GBM	734	0.60/0.59/0.60	Acc = 0.6 for RF
Lidaka 2015	Public	Internal (cross)	DSM-IV-TR	Functional MRI	Filter	PNN	640	-	Acc = 0.77 to 0.90, PPV = 0.78 to 0.88 and NPV = 0.77 to 0.92
Crippa 2015	Private	Internal (cross)	DSM-IV-TR	Behavior traits	Filter	SVM	-	-	Acc = 0.85 to 0.97
West 2014	Private	Internal (cross)	DSM-IV	Biochemical features	Embedded	DA (PLS)/SVM	61	0.92/0.95	Acc = 0.84/0.86
		Internal (split)	DSM-IV	Biochemical features	Embedded	DA (PLS)/SVM	21	0.81/0.84	Acc = 0.81/0.81
Wee 2014	Public	Internal (cross)	ADOS and ADI-R	Structural MRI	Others	SVM	117	1.00	Acc = 0.96
Price 2014	Public	Internal (cross)	DSM-IV-TR	Functional MRI	Filter(s)	SVM (single/multi-network)	60	-	Acc = 0.83/0.90

Uddin 2013	Private	Internal (cross)	ADOS and ADI-R	Functional MRI	Others	Regression (logistic)	40	-	Acc = 0.78
	Public	External	ADOS and ADI-R	Functional MRI	Others	Regression (logistic)	30	-	Acc = 0.83
Wang 2012	Others	Internal (cross)	ADOS	Functional MRI	Others	Regression (logistic)	58	-	Sens/Spec = 0.24 to 0.83/0.21 to 0.83
Wall 2012 (1)	Others	Internal (cross)	Algorithmic diagnosis	Behavior traits	Embedded	DT (ADTree, BFTree, CART or Decision Stump etc.)	627	-	Acc = 0.98 to 1.00
Wall 2012 (2)	Others	Internal (cross)	Algorithmic diagnosis	Behavior traits	Embedded	DT (ADTree, BFTree, CART or Decision Stump etc.)	996	-	Acc = 0.98 to 0.99
Calderoni 2012	Private	Internal (cross)	DSM-IV-TR	Structural MRI	Embedded	SVM	76	0.80	-
Jiao 2010	Private	Internal (cross)	DSM-IV	Structural MRI	Others	SVM	38	-	Acc = 0.71 to 0.76
				Structural MRI		MLP	38	0.78 to 0.80	Acc = 0.68 to 0.79
				Structural MRI		DT (functional trees or logistic model trees)	38	0.78 to 0.93	Acc = 0.71 to 0.84
Ecker 2010 (1)	Others	Internal (cross)	ICD-10	Structural MRI	Embedded	SVM	44	-	Acc = 0.68 to 0.81
Ecker 2010 (2)	Others	Internal (cross)	ICD-10	Structural MRI	Embedded	SVM	40	-	Acc = 0.45 to 0.90
Neeley 2007	Others	Internal	DSM-IV	Structural MRI	Others	DT (CART)	57	-	-

Abbreviation: DSM, Diagnostic and Statistical Manual of Mental Disorders; ASD, Autism Spectrum Disorder; TD, Typically Developing; ADOS, Autism Diagnostic Observation Schedule; ADI-R, Autism Diagnostic Interview-Revised; DNN, Deep neural network; SVM, Support vector machine; RF, Random forest; DT, Decision tree; PNN, Probabilistic neural network; DA, Discriminate analysis; EEG, Electroencephalography; Sens, Sensitivity; Sepc, Specificity; Acc, Accuracy; AUC, Area under the curve; Verbal ability, overall cognitive ability verbal ability, and non-verbal ability were measured by the Mullen Scales of Early Learning; PPV, Positive Predictive Value; NPV, Negative Predictive Value
